# Microarray Normalization Revisited for Reproducible Breast Cancer Biomarkers

**DOI:** 10.1155/2020/1363827

**Published:** 2020-08-06

**Authors:** Michael Kenn, Dan Cacsire Castillo-Tong, Christian F. Singer, Michael Cibena, Heinz Kölbl, Wolfgang Schreiner

**Affiliations:** ^1^Section of Biosimulation and Bioinformatics, Center for Medical Statistics, Informatics and Intelligent Systems (CeMSIIS), Medical University of Vienna, Spitalgasse 23, 1090 Vienna, Austria; ^2^Translational Gynecology Group, Department of Obstetrics and Gynecology, Comprehensive Cancer Center, Medical University of Vienna, Waehringer Guertel 18-20, 1090 Vienna, Austria; ^3^Department of General Gynecology and Gynecologic Oncology and Comprehensive Cancer Center, Medical University of Vienna, Waehringer Guertel 18-20, 1090 Vienna, Austria

## Abstract

Precision medicine for breast cancer relies on biomarkers to select therapies. However, the reliability of biomarkers drawn from gene expression arrays has been questioned and calls for reassessment, in particular for large datasets. We revisit widely used data-normalization procedures and evaluate differences in outcome in order to pinpoint the most reliable reprocessing methods biomarkers can be based upon. We generated a database of 3753 breast cancer patients out of 38 studies by downloading and curating patient samples from NCBI-GEO. As gene-expression biomarkers, we select the assessment of receptor status and breast cancer subtype classification. Each normalization procedure is applied separately, and biomarkers are then evaluated for each patient. Differences between normalization pipelines are quantified as percentages of patients having outcomes different for each pipeline. Some normalization procedures lead to quite consistent biomarkers, differing only in 1-2% of patients. Other normalization procedures—some of them have been used in many clinical studies—end up with distrusting discrepancies (10% and more). A good deal of doubt regarding the reliability of microarrays may root in the haphazard application of inadequate preprocessing pipelines. Several modes of batch corrections are evaluated regarding a possible improvement of receptor prediction from gene expression versus the golden standard of immunohistochemistry. Finally, we nominate those normalization methods yielding consistent and trustable results. Adequate bioinformatics data preprocessing is key and crucial for any subsequent statistics to arrive at trustable results. We conclude with a suggestion for future bioinformatics development to further increase the reliability of cancer biomarkers.

## 1. Introduction

### 1.1. Reliability of Microarray Gene Expression Measurements

Gene expression microarrays have been widely used to derive biosignatures for survival prediction [1] and allocating therapies in precision medicine [2, 3]. However, the reliability of such biosignatures has repeatedly been questioned [4, 5] and was targeted by an FDA-initiated Microarray Quality Control (MAQC) consortium [6]. Although microarray reliability was confirmed in some aspects [7, 8], the MAQC report found significant differences among research teams regarding the quality of data processing, and formulated the take-home message: “Classifier protocols need to be more tightly described and more tightly executed” [9].

Most of the above investigations assessed the quality of single microarray studies, each performed at some specific research center. Meanwhile, the Gene Expression Omnibus (GEO) [10] houses 110337 studies (as of March 12^th^, 2019), and reuse of this “big data treasure” has become not only a promising opportunity but also a significant challenge: Data from different studies not only pose additional questions about normalization but also show batch effects to be considered and properly adjusted [11–15]. Careful processing along reproducible pipelines is mandatory [16].

Given the availability of thousands of studies, the reuse of data offers an extraordinary wealth of a priori information, potentially fostering the conclusiveness of future studies. A mandatory precondition is, however, reusing data in a reliable fashion. Regarding microarrays, we have to envisage

- probe-specific biases and
- study-specific biases

overlaying and concealing each other. It is the ultimate goal of preprocessing pipelines to correct for both and render reliable estimates of gene expression, i.e., abundance of RNA transcripts.

We present a case study of pipelines for evaluating data processing quality for one of the most common expression arrays, the Affymetrix Human Genome U133 Plus 2.0 array, labelled “platform GPL 570” in GEO. We use 3753 samples from breast cancer patients out of 38 studies assembled from GEO [17].

It has been shown that different normalization pipelines influence gene-selection algorithms (and each one entails a specific bias-variance trade-off) [18]. Based on this, we critically compare normalization pipelines and available software implementations for joint evaluation of multiple gene expression studies and suggest procedural recommendations.

### 1.2. Normalization Methods for Single Studies

Microarray data acquisition is an intricate, multistep process [19], and adhering to standard operating procedures (tailored to the particular array) is mandatory for arriving at reliable results. In particular, microarray data need to be normalized before being analyzed, in order to compensate for biases inherent in measurement technology [20].

While early gene-expression chips could only perform comparative measurements (between two conditions: red/green), Affymetrix seemingly paved the way for absolute measurements by introducing mismatch (mm) probes in addition to perfect match (pm) probes. Each gene of interest is represented by several probe sets, most of them capturing a specific part of a gene's transcript. Several algorithms have been proposed to aggregate estimates from single probes to an estimate for a whole probe set, finally to be stored in CEL-files. All in all, the Affymetrix Human Genome U133 Plus 2.0 chip holds 54675 probe sets, out of which 62 are control, and the remaining 54613 represent a total of 23035 genes named in HUGO. They tailored normalization algorithms MAS4 and MAS5 [21], subtracting unwanted, nonspecific-hybridization to mm-probes from pm-probes. The aim was to enable absolute measurements of gene expression on a totally new and reliable basis. A MAS5-implementation in R is freely available [22]. However, as early as 2002, physical analysis of hybridization [23] explained what was seen before in measurements: up to 30% of mm-probes yield intensities even higher than their pm-counterparts, resulting in (obviously unrealistic) negative intensities upon subtraction [24]. This indicates that elaborated background correction may improve accuracy but, in general, worsens precision [25]. The MAS5 algorithm circumvents this failure by ad hoc corrections (reviewed in [26]). In 2004, Affymetrix released a new algorithm, PLIER (Probe Logarithmic Intensity Error), also considering pm and mm-probes [27]. However, ultimately neither one succeeded in establishing itself as a golden standard. Instead, the bioinformatics community continuously improved other normalization algorithms as possible remedies, as reviewed by ([24, 28]. They all disregarded mm-probes, the very asset of Affymetrix. Robust Multi-array Average (RMA), performed a background correction on statistical grounds rather than mismatches, was implemented within the “affy” package in R (Irizarry, [24]), and finally recommended even by Affymetrix. It has since become the most widely used normalization algorithm worldwide [22].

Since high promises put into microarrays were repeatedly hampered by substantial difficulties, it is no surprise that microarray normalization has become a key issue of bioinformatics research [29], including benchmark comparisons between normalization procedures [30, 31] and possible batch corrections [12, 13, 25, 32–34]. Later on, RMA also appeared in a MATLAB implementation.

To shed light on this intricate situation, we demonstrate normalized expression values to differ significantly between R and MATLAB, even in their distribution, and yet more in values themselves.

Later on, the algorithm GCRMA was developed (Wu and Irizarry, [35–39]) to correct for hybridization bias due to the higher binding energies of GC base-pairs as compared to AU pairs. Indeed, we found remarkable differences in results between RMA and GCRMA. Surprisingly, only few researchers in the community have adopted GCRMA, despite its conceptual superiority. What are the reasons? We shall present a possible explanation.

A fairly recent development is IRON (Iterative Rank Order Normalization) [40], combining advantages of RMA and MAS5, disregarding mm-probes, however. The procedure fRMA [41] is another option.

Finally—and most importantly—the question arises, to which extent biomarkers derived from expression data might be more or less unstable, due to different normalization pipelines. Are biomarkers based on microarray data reliable at all? We shall provide a case book of examples.

### 1.3. Combining Multiple Studies

Data reuse as well as meta-studies need to jointly evaluate multiple gene expression studies (Studies are called “series” in GEO.) [41]. This poses additional questions, some of which have been addressed by the batch-correction method “Combat” [12–14, 33] as well as by “surrogate variable analysis (SVA)” [42–44]:

1. Should each study be normalized on its own (token “single” in this work), then normalized data be combined and finally batch effects be removed?
2. Or else, should samples of multiple studies be jointly normalized (token “global” in this work) and batch effects eliminated thereafter?
3. Or may global normalization even by itself remove batch effects, rendering a subsequent batch correction unnecessary?

Data used in this work have been obtained and preprocessed as described in subsection “Data used”. We evaluate the performance of normalization pipelines as detailed below and conclude with recommendations.

## 2. Material and Methods

### 2.1. Data Used

For the present study, 3753 Cel-files and attached (clinical) data (characteristics) were downloaded from Gene Expression Omnibus (GEO) [10] and curated as follows:

1. To keep technology homogenous, only platform GPL570 was considered
2. Since we focus on breast cancer tumor samples, control samples were excluded, see column “Control” in Table 1
3. Replicates (several measurements on the same patient/biosample, as marked in GEO) were removed (column “Replicates” in Table 1)
4. Duplicates (equal CEL-files in different GSE-series) often are given a new GSM-number, concealing the fact that they are duplicates. They cannot be detected by simple comparison, due to differences in CEL-file formats and in container packing. We hence resorted to pairwise comparison of extracted expression values, and then excluded duplicate samples
5. In case metadata were inconsistent between duplicates, these were curated manually
6. Furthermore, we identified all GSE-series which contain not only novel samples but also samples from previous series included as duplicates. Such duplicates are prone to create heterogeneity within the series to which they have been added (see section “Explicit batch correction”). We therefore left them with their original series (and deleted their duplicates), see column “Duplicates” in Table 1
7. Damaged samples were excluded, based on inspection using RMAexpress [45], see column “others” and “comment to others” in Table 1
8. We inspected within-series heterogeneity. Already simple PCA indicates that samples within some studies have been processed along different protocols, e.g., GSE32646, GSE47109, and GSE50948. Inspection of protocols reveals FFPE probes, see column “others” and “comment to others” in Table 1
9. Outliers, i.e., samples with data seemingly different from others, have been retained, provided they are technically ok

Finally, we end up with 3753 samples being used out of 38 series, see column “samples used” in Table 1. The file “sample.csv” in supplementary material lists each sample used in the present work. The following metadata were included from GEO: sample-ID (GSM-number) and allocated study number (GSE-number) CEL-filename, source_name_ch1, and title. Further metadata have been extracted from GEO and relevant publications (estrogen receptor status (ER), progesterone receptor status (PGR), and HER2 status (HER2). Values are given only if these data have been measured (by IHC, FISH, etc.), not merely computed or imputed. Furthermore, values were set to missing (not a number, NaN) in cases of not fully informative data within papers and GEO or if duplicate samples contained contradicting information.

The file “annotations.csv” in supplementary material gives mapping information from Affy probe-set-id to HUGO gene names.

We consider breast cancer studies only with platform GPL570 from GEO. Since we focus on breast cancer tissue, “control” samples were excluded (see column “control”). Out of those “Samples in GEO”, we excluded samples according to several criteria (see subcolumns of “Criteria for excluding samples” and ended up with “nr of samples used” in this work. For details on data curation, see section “data used”. Within each study, samples are sorted according to ascending GSM numbers and a sample-ID (column in table) was created. Finally, whole studies were sorted by descending number of samples and then listed in the table. Please note that GSM781392 (index 1711) and GSM2345373 (index 3713) are identical samples.

### 2.2. Data Processing Pipelines

The description of methods in this section is illustrated by specific examples appearing in tables and figures. These not only illustrate the methods being explained but also anticipate parts of the results presented later in detail (We preferred this structure against strictly separating results from methods to avoid redundancies.) (to avoid redundancy in the results section). Note that, starting with Table S1 and Figure S1, the material is shown in supplementary materials.

**Figure 1:**
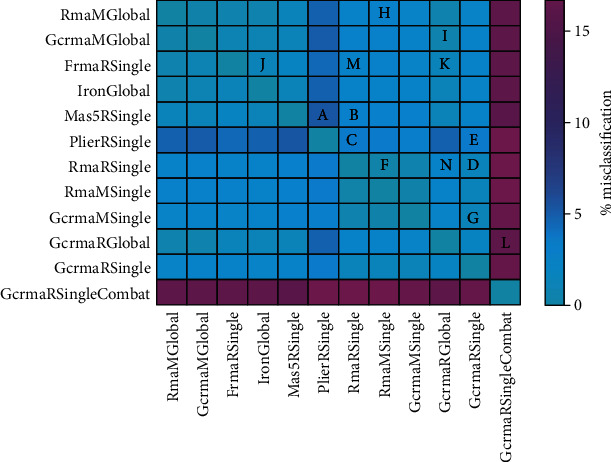
Comparison of pipelines. Each pipeline is given a token (e.g., RmaMGlobal) for reference throughout the paper. Specific pairs of pipelines have been selected to be compared in detail regarding the differences in output they produce. Each of these comparisons is labelled by a capital letter (“***A***” to “***N***”) for later reference. Note that only a reasonable selection (and not all possible) comparisons have been considered in detail. Moreover, all possible comparisons are quantified by the underlayed heat map, giving the percent misclassification in prediction of oestrogene receptor status, see the colorbar on the right. This heat map shows the values given in the first line (ER) of Table 2.

Each “data processing pipeline” receives the same input from GEO and performs data cleansing (which is identical for all pipelines considered) as described in section “Data used”. Cleansing is followed by normalization and batch correction. The latter is performed optionally, if appropriate. We investigate and compare two possible methods, ComBat and SVA, the latter in several modes. However, only ComBat after one specific normalization (GCRMA) is included in the set of pairwise comparisons for reasons of multiplicity. We label each “pipelin” with a token for later reference, when describing results in a case book.  Figure 1 shows pipelines, labels, and comparisons (“***A***”, “***B***”,…, “***N***”) between pipelines which have been examined.

### 2.3. Comparing the Results for Pairs of Pipelines

#### 2.3.1. Statistical Metrics

In order to compare pipelines regarding differences in outcome, we provide 4 statistical metrics, each based on all 54675 probe sets.

**Figure 2:**
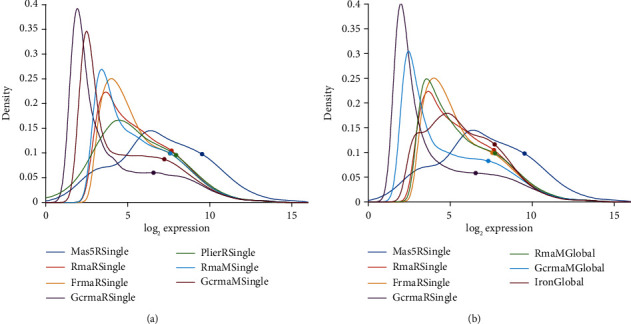
Probability distribution of expression values after different normalizations.

**Figure 3:**
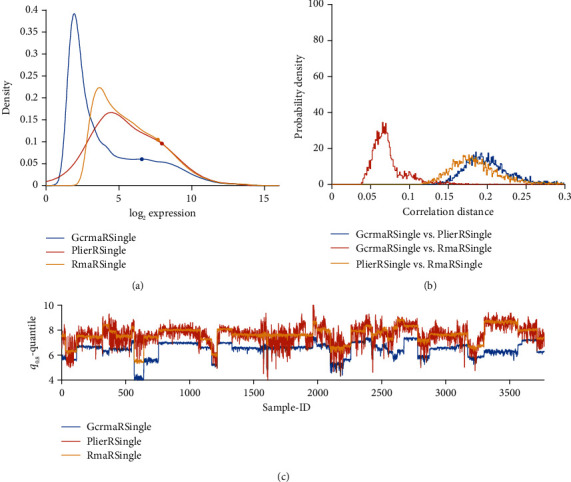
GCRMA versus PLIER, compared with RMA, applied to single studies. (a) Distribution profiles of log_2_-normalized expression values. (b) Histogram of correlation distances of log_2_-normalized expression values between pairs of pipelines. (c) For all 54675 probe sets of each sample, the 80% quantile (***q***_0.80_) is computed. Then, ***q***_0.80_ quantiles are plotted over sample-ID (*x*-axis).

**Figure 4:**
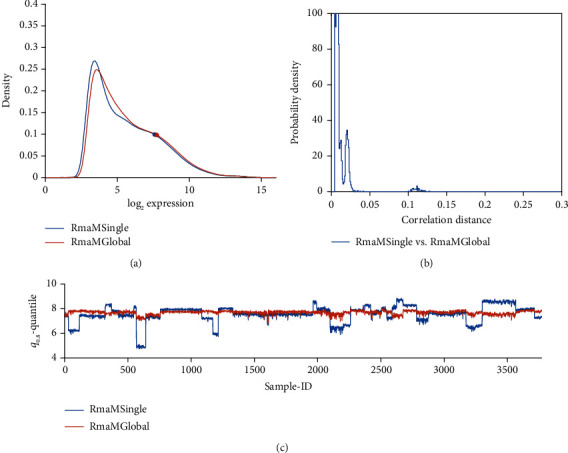
RMA single versus RMA global, both in MATLAB implementation (comparison “*H*”). (a) Distribution profiles of log_2_-RMA normalized expression values. (b) Histogram of correlation distances of log_2_-normalized expression values between pairs of pipelines. (c) For all 54675 probe sets of each sample, the 80% quantile (***q***_0.80_) is computed and plotted over sample-ID (*x*-axis).

**Figure 5:**
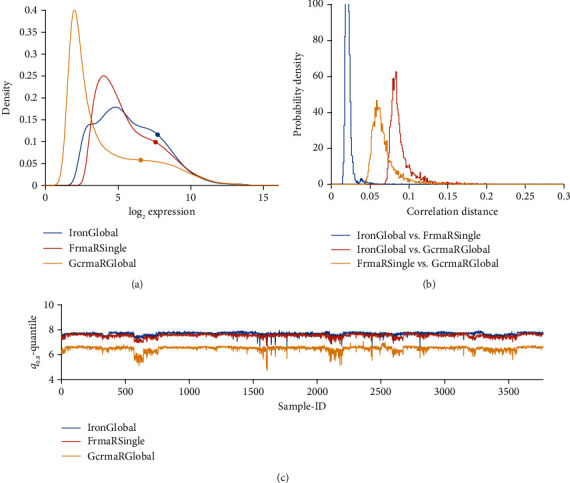
IRON global vs. fRMA (Comparison “*J*”) and fRMA vs. GCRMA R global (comparison “*K*”). (a) Distribution profiles of log_2_-RMA normalized expression values. (b) Histogram of correlation distances of log_2_-normalized expression values between pairs of pipelines. (c) For all 54675 probe sets of each sample, the 80% quantile (***q***_0.80_) is computed and plotted over sample-ID (*x*-axis).

**Figure 6:**
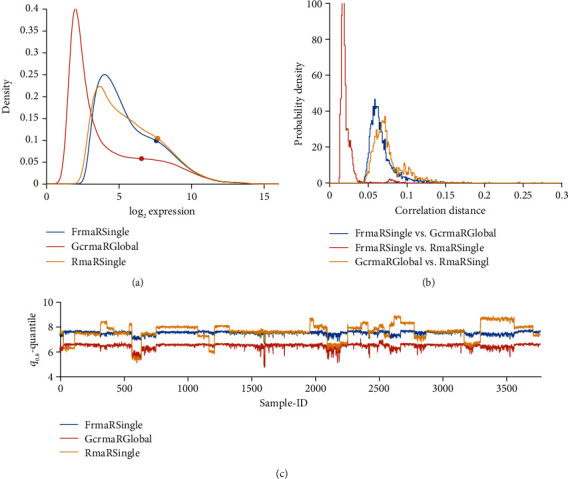
Comparison between the 2 top pipelines, fRMA, and GCRMA. For reference, we also show the “dinosaur”, “RMA R single”. The derby “fRMA vs. GCRMA R global” is labelled comparison “***K***” in Table 2 to Table S1. Comparison of fRMA with the dinosaur “RMA R single” is labelled “***M***”. “GCRMA R global” versus “RMA R Single” is labelled “***N***”. (**a**) Distribution profiles of log_2_-RMA normalized expression values. (**b**) Histogram of correlation distances of log_2_-normalized expression values between pairs of pipelines. (**c**) For all 54675 probe sets of each sample, the 80% quantile (***q***_0.80_) is computed and plotted over sample-ID (*x*-axis).

**Figure 7:**
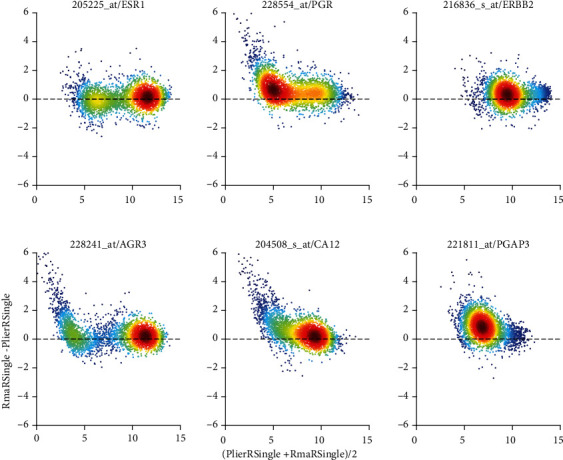
PLIER vs. RMA, applied to single studies (Comparison “*C*”) via Bland-Altman plots of log_2_-expression values. Bias correction by PLIER seems to generate linear trends towards low expression values. *x*-axis label is short for [log_2_(PLIER) + log_2_(RMA)]/2 and *y*-axis label is short for log_2_(RMA)-log_2_(PLIER). Note that differences and sums of logs represent ratios and products of raw expression values, respectively. All Bland-Altman plots of this paper comply with this short-form of notation. Left column: two probe sets most relevant for representing the estrogen receptor (205255_at: gene and 228241_at: cogene). Middle column: progesterone receptor (228554_at: gene and 205225_at: co-gene). Right column: Her2 (216836_s_at: gene and 221811_at: co-gene). Colors code density of values: dark red for high density, dark blue for low density.

**Figure 8:**
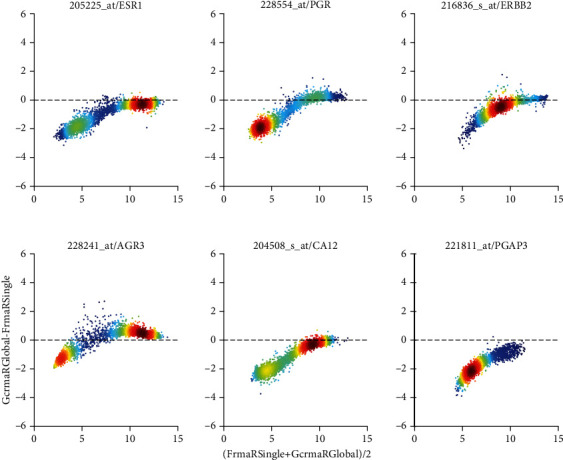
fRMA vs. GCRMA R global (comparison “*K*”), shown as Bland-Altman plot. For details regarding panels and axes, see caption of Figure 7.

**Figure 9:**
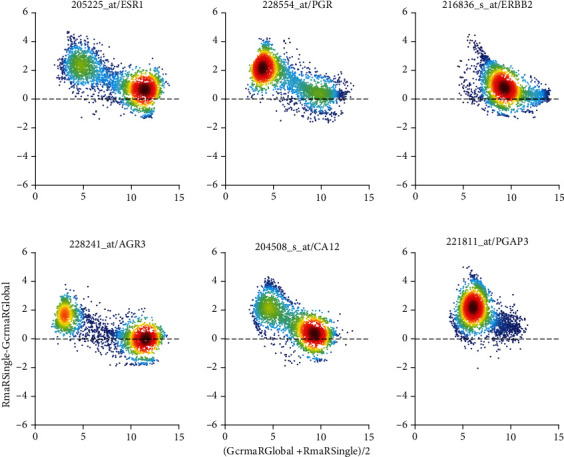
“GCRMA R global” versus “RMA R single” shown in Bland-Altman plot (comparison “*N*”). The bimodal distribution of values corresponds to receptors being “not expressed” (low values) versus “expressed” (large values). Note that small values are to a large extent buried in noise. For details regarding panels and axes, see caption of Figure S1.

1. Probability distribution of normalized probe values, separately for each pipeline. For an overview, see Figure 2. Distribution profiles have been obtained via kernel methods based on normal distribution [46, 47], rather than via simple histograms. Kernel methods yield reliable estimates, even in case of data artifacts. For specific, pairwise comparisons see panels (a) in Figures 3–6 and Figure S4 (in results section or supplementary material, respectively).
2. Frequency distribution of correlation distances for the particular comparison between pipelines, see the histograms in panels (b) of Figures 3–6 and Figure S4. Note that lower correlation distance indicates more similarity between the results of two pipelines. A (maximum) distance of 1 indicates no relation at all. For mathematical details on distances, see the Supplementary Material
3. The 80%-quantile, ***q***_0.80_, is computed from all 54675 probe sets of each sample. We adopted the 80%-quantile (rather than the 50%-quantile, i.e. median) as representative expression value for each sample. Given that ***q***_0.80_ is quite rough, a measure to characterize all probe sets of a sample, it will certainly fail to reveal intricate relations between single genes or even between groups of genes. However, it serves well for an overview. Samples were numbered consecutively through all studies, see Table S5 and panels (c) in Figures 3–6 and Figure S4.
4. For selected pairwise comparisons, we additionally display Bland Altman—plots [48] showing detailed differences in outcome for selected probes [20], see Figures 7–9 and Figure S6.

Kernel-density estimators [46, 47] based on a normal distribution have been used to generate these probability density plots. Note that kernel estimators suppress outliers. Log_2_ expression on the *x*-axis actually stands for log_2_ fluorescence [49] (in this and all subsequent figures). GCRMA-normalization yields the smallest mode and discriminates best between expressed and nonexpressed probe sets. Profiles after applying Combat almost coincide, although correlation is weaker.

#### 2.3.2. How Different Pipelines Affect Biomarkers

Distances and correlations are sound mathematical concepts. However, for precision medicine, it is even more relevant if biomarkers result stable and reproducible: Do biomarkers, as determined from a given array (i.e., sample), lead to unique consequences for a given patient, irrespective of the particular pipeline being used? To clarify, we compute two test sets of predictive biomarkers:

1. Prediction models for 3 hormone receptor status (ER, PGR, HER2) in breast cancer patients, as published earlier by our group [17, 50]. In the present work, we apply the very same prediction algorithm (“odds”-method) on expression data normalized in different ways
2. 6 different breast cancer subtyping algorithms (“biomarkers”: smgene, scmod1, scmod2 pam50, ssp2006 and ssp2003)), rendered by the well-known, publicly available “genefu” software package [50, 51].

Regarding (1), we predicted receptor status, yielding one out of 3 classes—positive, negative, or unknown—for each of the receptors (estrogen (ER), progesterone (PGR), and HER2. For a given comparison (say “***A***”, compare Figure 1) between pipelines, we count the percentage of patients with different outcome between pipelines. For example, Estrogen (ER) status was predicted differently for 2.9% of patients between pipelines “RMA Matlab global” and “RMA Matlab single” (comparison “***H***”), see Table 2.

Regarding (2), patients are divided in either 4 classes (via biomarkers smgene, scmod1, scmod2) or in 5 classes (via biomarkers PAM50, ssp2006, ssp2003). For each class, a specific treatment is adequate and misclassification may significantly reduce survival chances or unnecessarily increase unwanted side effects. The difficulty and yet focal importance of adequate preprocessing of data has been deplored in a seminal paper [51]: “The gene expression datasets taken from public databases and websites were not normalized”. We assume that, due to the lack of trustable normalization pipelines, genefu had to be established on nonnormalized data.  Table 3 shows, however, that normalization has a significant impact on class prediction.

First, all arrays are processed by each of the two pipelines to be compared. Then, for each array (out of 3753), all biomarkers are evaluated to see how much these predictions differ due to preprocessing via different pipelines. For each biomarker, we give percentages of patients for which this biomarker would change (percent misclassification, see Table 2) and additionally provide Cohen's kappa [52] in Tables S1 and S2. Note that, when referring to “biomarkers” in this article, we implicitly refer to the above sets (1) and (2).

## 3. Results

All in all, we have included 12 pipelines in pairwise comparisons. Out of all possible pairwise comparisons (6∗11 = 66), we present a small selection to highlight differences of particular interest regarding approach, performance, stability, or results. More comparisons are shown in the supplementary material.

Secondly, and maybe even more interestingly, we evaluate the performance of single pipelines in predicting receptor status against the golden standard IHC. Since some “ground truth” is at hand in this case, we are able to compute “percent misclassification” as a measure of performance.

RMA in R implementation, being the most widely used pipeline, appears in many of our comparisons and evaluations. On a visual basis, all possible pairwise comparisons are summarized as heat map in Figure 1.

In the following detailed description, we let—for ease of reference—comments immediately follow the corresponding results, rather than relegating them into the discussion section.

### 3.1. Considering Mismatch-Probes for Probe-Specific Bias Correction: MAS5 And PLIER

**Figure 10:**
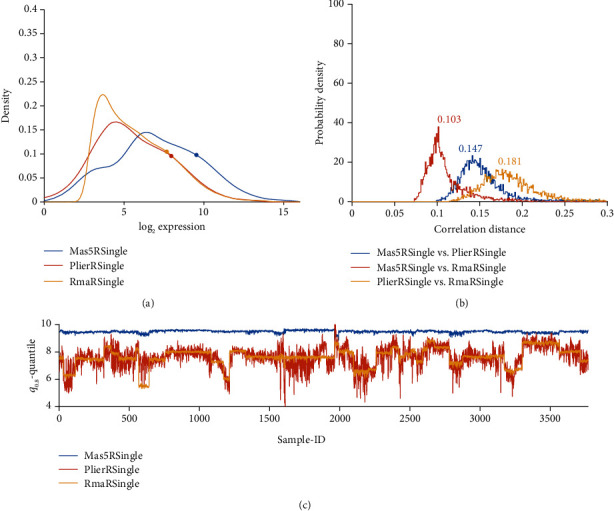
Effect of bias correction via mismatch probes: MAS5 versus PLIER versus RMA, applied to single studies. (a) Distribution profiles of log_2_-normalized expression values of all 54675 probe sets (normal distribution kernel method). 80%-quantiles, ***q***_0.80_, are shown as solid circles. (b) Histogram of correlation distances of log_2_-normalized expression values between pairs of pipelines. e.g., for the comparison MAS5Single vs. RmaRSingle (red histogram, labelled comparison “***B***” in Figure 1 and Table 3): All samples are normalized by MAS5RSingle and also by RmaRSingle, and then examined in pairs: Within each pair, the correlation distance is computed from all 54675 probe sets (see text and caption of Table 3). The frequency distribution of distances is shown as a histogram. These distributions are quantitatively characterized by mean and quantiles (median, ***q***_0.15_, ***q***_0.85_) in Table 3. For transparency, we show the median value also aside the histogram. Panel (c): For all 54675 probe sets of each sample, the 80% quantile (***q***_0.80_) is computed. This is done for each of the 3753 samples and plotted over sample-ID (*x*-axis). Sample-ID runs consecutively over all 38 studies. The overall ***q***_0.80_ (over all probe-sets and samples) is marked as full circle in the expression profile of each pipeline in panel (a).

As the first pipeline, we define MAS5 applied to single studies, using the R implementation (pipeline token “MAS5 R Single”). As the second pipeline, we define PLIER using the Bioconductor R implementation (token “PLIER R Single”), see Figure 10. This particular comparison is labelled “***A***” in Figure 1 and all other tables, see below. For reference, we also show results of the world's most widely used normalization, RMA (R-implementation), applied to single studies (RMA R Single).

#### 3.1.1. Statistical Criteria for Differences between Pipelines

Figure 10 compares expression profiles of all 54675 probe sets (panel (b)), 80%-quantiles of probe sets for each of the 3753 samples (panel (c)), and the histogram of correlation distances between pairs of samples, panel (b).

Results differ substantially: MAS5 yields a distribution centered at larger expression values (panel a) and is little affected by study-specific bias, panel (c). PLIER shows considerable variation within studies, comparable to variation between studies, panel (c). This is in contrast to all other pipelines, see below. We may interpret this as follows: PLIER preserves biologic variation within studies in such a way that it is not dominated by batch effects between studies. In contrast, MAS5 gets rid of all batch effects but at the same time might be prone to extinct also biological variation. Numerical results for correlation distances are given in Table 3.

Pairwise comparisons between data processing pipelines via distance distribution statistics. Pairwise comparisons of pipelines are labelled by “***A***”–“***N***”, e.g. “***A***” standing for the comparison “MAS5 R Single” versus “Plier R Single”, see Figure 1. Distance between samples after 2 different normalization pipelines was defined via Pearson correlation (rho) computed between corresponding probe sets (54675). We show the mean distance $\bar{d}=1-\rho$ over 3753 samples together with quantiles (***q***_0.15_ to ***q***_0.85_). Smaller values indicate more similar results. Note that these quantiles describe specific points in the distributions shown in panels (b) of Figure 4, Figure 5, Figure 6, Figure 10 and Figure S4.

Both, MAS5 and PLIER, use mm-probes designed by Affymetrix to remove hybridization bias. After all, this was considered the stronghold of Affymetrix in paving the way towards absolute measurement of gene expression. This is most obvious in Bland-Altman plots, see Figure 7 and Figure S2. We display pairs of probe sets found relevant for the estrogen receptor (left column), the progesterone receptor (middle column), and the HER2-receptor (right column) [50].

RMA was established to overcome MAS5's subtraction of mismatch intensities, considered unrealistically large. This came with the cost of replacing probe-specific bias correction by a blunt estimate. In fact, RMA deviates significantly from MAS5 (comparison “***B***”): We find systematic differences in absolute values, and these differences also depend on the mean in a suspicious way, as clearly visible in Figure S2: MAS5 subtracts increasingly more bias as expression values are large. Can such a correction safely be ignored by RMA? This difference is clearly revealed also in Figure 10, panel (a). It is interesting to remark: Although RMA ignores a key feature of the Affymetrix array design; it became the most frequently used normalization pipeline worldwide.

Considerably later, PLIER was designed to overcome the weaknesses of MAS5 and RMA by reviving consideration of mismatches. Figure 7 shows this comparison, labelled “***C***”, for the probe sets of receptor genes and cogenes. Systematic differences in absolute values are close to zero. However, for low expression values, distinct trends are visible, most probably relating to different probe-specific bias corrections performed by the two pipelines. However, these discrepancies occur at such low expression values that they—despite looking spectacular—in fact mean nothing but discrepancies in noise.

Finally, see comparison labelled “***A***”, MAS5 versus PLIER, in Figure S1. Both normalizations consider mismatches. No suspicious trends can be observed in the differences but considerable spread in relative differences (-5 to +5 in log values) is obvious. PLIER values are systematically below MAS5 values.

#### 3.1.2. Robustness of Receptor Status when Pipelines Are Swapped

Now we turn to the stability of biomarker predictions as pipelines are swapped, see columns “***A***” to “***N***” of Table 2 to Table S1, respectively. Each column represents the swap between a specific pair of pipelines, e.g., “***A***” stands for “MAS5 R single” versus “PlierRSingle”, and we start with the biomarker “hormone receptor status prediction”. Numbers represent estimates of the discordance between pipelines, expressed as “percent misclassification” (Table 2) and Cohen's kappa, respectively (Table S2). The first 3 comparisons, already mentioned above (***A*, *B*, *C***) within the triple {MAS5, RMA, PLIER}, are in bold entries (To contrast them against the other comparisons discussed below. Nevertheless, the whole table (also including all other comparisons) is shown here for ease of reference.) to contrast them from comparisons anticipated in the table here, but described later.

Interestingly, despite excellently looking BA-plots (Figure S2), MAS5 and RMA (comparison “***B***”) yield fairly discordant receptor status (8.1% misclassification on average). In contrast, mm-probes disregarded by RMA does not seem to severely harm (as compared to MAS5 or PLIER, columns “***B***” and “***C***”), ending up with relatively low misclassification (5,9%, 5,7%). Table S2 shows corresponding results in terms of kappa.

Pairwise comparisons (“***A***”–“***N***”) between pipelines, see Figure 1. Note that each estimate may assume one of 3 states (positive, negative, indefinite) as indicated in column #. Numbers in the table give the percent of patients showing any of 3∗(3 − 1)/2 = 3 possible disagreements in result. Note that this table comprehensively shows all comparisons between pipelines discussed in this work. The line “average” summarizes individual percentages of 3 receptors for each comparison. Note also that these values appear color-coded in Figure 1, which in addition gives estimates for all other possible comparisons of pipelines.

#### 3.1.3. Robustness of Breast Cancer Subtype Prediction when Pipelines Are Swapped

Table 4 shows differences in the six biomarkers for “breast cancer subtype prediction” (output of the “genefu” software package [53]) when swapping normalizations. Not surprisingly, the smallest average difference (1.5%) is seen between different software implementations of the same normalization (RMA) in R versus MATLAB, column “***F***”. However, striking differences are seen between the conceptually different methods MAS5 and PLIER (22.3%, column “***A***”), GcrmaRGlobal and GcrmaRSingleCombat (22.9%, column “***L***”), PLIER and RmaRSingle (23.2%, column “***C***”) as well as between PlierRSingle and GcrmaRSingle (25.4%, column “***E***”). Note that in particular the first biomarker of “genefu”, “smgene”, proofs excessively sensible to swaps “***C***” and “***E***”, yielding discrepancies up to almost 60%.

We evaluate six biomarkers computed by algorithms within the R-package “genefu”: scmgene, scmod1, and scmod2 assign subtypes out of 4 classes, whereas the other 3 assign subtypes out of 5 classes, see column “#”. For each specific comparison (labels “***A***”–“***N***”, see Figure 1) between 2 pipelines, we show the percentage of patients who would be assigned different subtypes if genefu was performed after each of the pipelines.

Table S1 shows corresponding results in terms of kappa. Note misclassifications according to Cohen's kappa differ in ranks as compared to “percent misclassification” (Table 4). Again “smgene” proofs exceptionally sensible, yielding even negative kappa for “***C***” and “***E***”. We note that in each of these poor comparisons, PlierRSingle is involved.

### 3.2. Probe Specific Hybridization Bias due to GC-Content

Probes rich in GC pairs bind stronger than others, inducing a positive bias of estimated expression. GCRMA was designed to compensate this bias by reducing intensities of respective probes. As a result, normalized intensities of many probes are shifted towards lower values, see the frequency distributions Figure 3. GCRMA renders a significant number of probes quasi “non-expressed”, while approving a smaller portion of “vigorously expressed” probes for further analysis, thereby likely to increase the reliability of results.

Alternatively, PLIER disregards GC content but considers mismatch probes like MAS5, see above. Hence both, GCRMA and PLIER, correct hybridization bias specifically for each probe, while RMA performs only a blunt bias correction. It seems questionable if the intricate correction efforts of GCRMA and PLIER are actually rewarding as compared to RMA, which bluntly ignores mm-probes and base-pair composition.

GCRMA yields expression values significantly lower in all studies and shows study-batch effects similar to RMA: within studies, variability is much lower than between studies. Opposed to this, PLIER yields considerable variability even within studies, comparable in magnitude with variation between studies. As a result, batch effects appear less pronounced.

#### 3.2.1. Statistical Metrics

**Figure 11:**
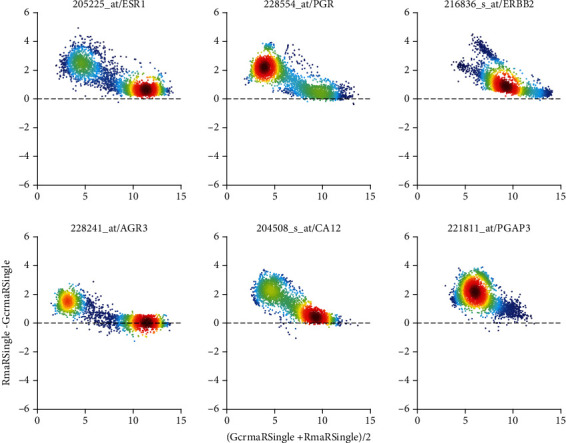
GcrmaRSingle vs. RmaRSingle (Comparison “*D*”) via Bland-Altman plots of log_2_-expression values. For details regarding panels and axes, see caption of Figure 7.

GcrmaRSingle differs from RmaRSingle considerably on probe set level, see Figure 11. While probe sets for the estrogen receptor (left column) show only a general bias (RMA > GCRMA), probe sets for progesterone (middle column) and for Her2 (right column) exhibit distinct linear correlations—most likely reflecting the subtraction of hybridization bias, which is assumed to increase with gene expression. As a result, GCRMA-normalized values decline far below RMA-normalized ones, as expression increases. Finally, it is interesting to see how GCRMA and PLIER compare, after all both perform probe-specific bias correction—in different ways, however, see Figure S3. Differences strikingly depend on the magnitude of values.

#### 3.2.2. R versus Matlab

Different implementations (R vs. MATLAB) of the same algorithm should be expected to yield equal results. However, for GCRMA, we find distinct differences, see section 7.4 in supplementary material.

### 3.3. Normalization of Multiple Studies

To the best of our knowledge, all normalization methods mentioned above have originally been devised for application to single studies (in this work labelled by token “single”). If multiple studies were to be analyzed, in many cases, “meta-studies” were performed [54]: Separately for each study, relevant biologic information was extracted (e.g., biomarkers computed) and these information then merged (“late merging”). It was also common practice to normalize values for single studies and then aggregate normalized values for joint analysis [55]. In contrast, multiple studies with expression values may be concatenated and special batch corrections applied to in order to reduce study bias [12, 32]. From these batch-corrected expression data, biomarkers may then be derived.

An approach intentionally reducing study bias in multistudy normalization has been undertaken by IRON. It first generates a “reference chip” from all arrays to be analyzed and then normalizes all chips according to that reference. IRON is thus designed to handle multiple studies as one single batch.

A similar but even more general approach is realized by “frozen RMA” (token “fRMA”): It computes a reference profile of gene expression from samples of the respective platform in GEO, and based on that performs RMA [41], separately for each sample submitted. It thus allows normalization of small numbers of additional samples—e.g., from incoming patients, independent of samples already normalized before. In a sense, fRMA can be seen as a generalized form of RMA.

As an additional alternative, we follow the proposal to normalize samples from several studies collectively [16, 56] as if it were a single dataset (“early merging”, labelled by token “global” in this work) and demonstrate the effect. Applying RMA to multiple studies seems to simultaneously remove batch effects due to study bias, even though RMA was not designed with that purpose in mind.

The same holds for GCRMA: It almost completely eliminates study-bias, in addition to its merits regarding probe-specific bias reduction.

Note that fRMA is exceptional: To the best of our knowledge, it is the only normalization method yielding unique results for a given chip, i.e., “fRMA Sinlge” is in effect identical to “fRMA Global”.

#### 3.3.1. RMA Applied to Multiple Studies

**Figure 12:**
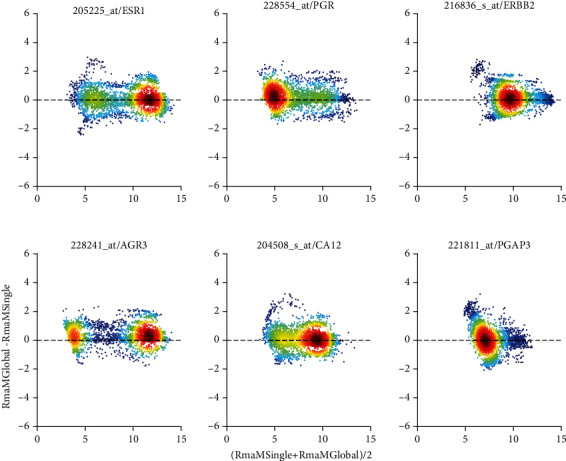
RMA global versus single, implemented in MATLAB (Comparison “*H*”) via Bland-Altman plots of log_2_-expression values. For details regarding panels and axes, see caption of Figure 7.

We first investigate the consequences of applying RMA not only to single studies but simultaneously to a whole bunch of studies: The comparison “RMA M Single” versus “RMA M global” is labelled “***H***” in Figure 1; for results, see Figure 4: Expression profiles (a) look similar, correlation distances (b) small, but “RMA Single” leaves us with severe batch effects due to study bias. “RMA M global” seems capable of removing these batch effects, at least as far as they are reflected by ***q***_0.80_. Pairwise comparisons of normalized values are shown in Figure 12.

Unfortunately, however, it was technically impossible for us to normalize all 3753 samples jointly in R, seemingly due to weaknesses in software design, rendering “RmaRGlobal” unfeasible. The cause of this problem was a memory leak (‘memory not mapped'). Even when executing Bioconductor rma on a machine with excessively larger memory (256 GB), the same type of error occurred. We performed bench marks with increasing numbers of samples and found an approximate limit of 2K samples. This number is to be interpreted with caution, since input (CEL) files vary in size (even for the same chip), depending on the format. Moreover, the same error occurs with any of the derivative procedures (RMAexpress, fRMA, justRMA,…) based on the same C++ sources. For machine specifications, see also caption to Table S5.

For global RMA, we instead had to retreat to MATLAB, presuming the bioinformatics community up to now could not benefit from global RMA normalization of large joint studies (given the fact that R is predominantly used in bioinformatics as compared to MATLAB, also due to costs).

#### 3.3.2. GCRMA for Multiple Studies

While the R-implementation of RMA (for technical reasons) cannot normalize our set of samples in a single batch, the R-implementation of GCRMA, surprisingly, can. We have to note, however, that careful setting of parameters is mandatory.

**Figure 13:**
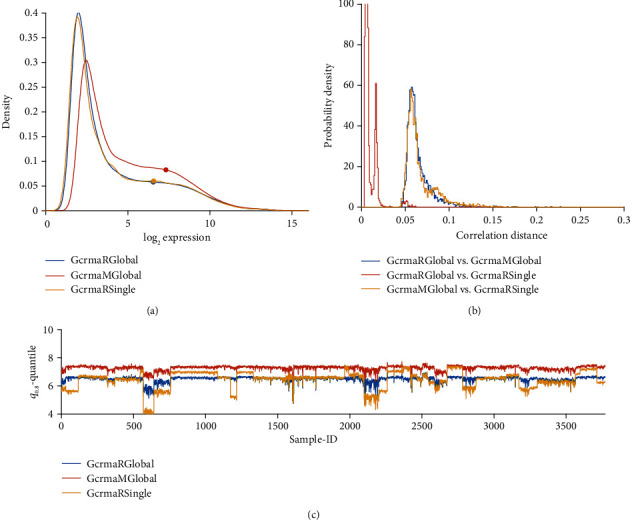
Comparing several flavors of GCRMA: GCRMA R global—GCRMA M Global—GCRMA R Single: (a) Distribution profiles of log_2_-RMA normalized expression values. (b) Histogram of correlation distances of log_2_-normalized expression values between pairs of pipelines. (c) For all 54675 probe sets of each sample, the 80% quantile (***q***_0.80_) is computed and plotted over sample-ID (*x*-axis).

**Figure 14:**
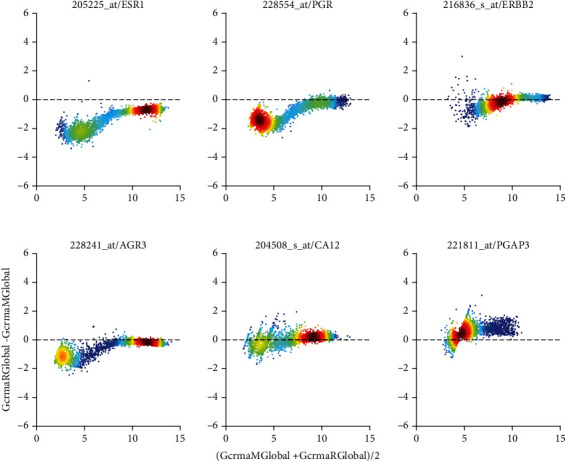
GCRMA R global vs. GCRMA Matlab global, shown as Bland-Altman plot. For details regarding panels and axes, see caption of Figure 7.

We compare the pipelines “GCRMA R global” with “GCRMA Matlab global” and include “GCRMA R Single” as reference for existing study-specific bias, see Figure 13. Distributions of normalized values are very similar and correlation distances low between GCRMA applied to single studies versus all studies. Similar to RMA, also for GCRMA, the R implementation yields consistently lower values than MATLAB does. Batch effects visible after GCRMA-normalization of single studies are drastically reduced by “global” normalization. This holds for R as well as MATLAB. For a comparison on a probe-set basis, see the Bland-Altman plot in Figure 14.

#### 3.3.3. Frozen RMA and IRON

Efforts to extend RMA by design towards multistudy normalization resulted in and fRMA [41] and IRON [40].

Frozen RMA (fRMA) allows one to analyze samples individually or in small batches and then combine the data for analysis. This is achieved by drawing on publicly available databases to estimate probe-specific effects and variances (frozen estimates). These frozen parameters are used to normalize any new array. The fRMA is particularly useful when it is not feasible to preprocess all of the data simultaneously.

FRMA and IRON base normalization on unified expression profiles and drastically reduce study bias, see Figure 5. IRON derives a unified profile from the set of samples submitted, and hence normalization-results for the individual sample depend on this set of samples submitted. As opposed, fRMA truly yields the same normalized data for a given sample, independent of all other samples submitted.

**Figure 15:**
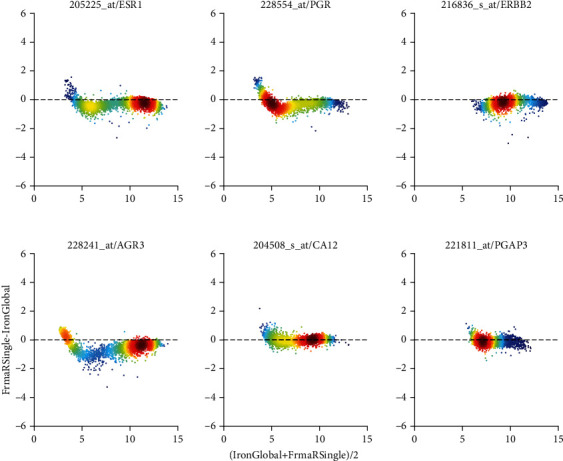
IRON vs. fRMA, jointly applied to all studies (Comparison “*J*”), shown as Bland-Altman plot. For details regarding panels and axes, see caption of Figure 7.

The differences between IRON global and fRMA regarding individual probe sets are shown in Figure 15. Only minor trends are visible, indicating that both pipelines yield fairly comparable results. Predictions for hormone receptors differ by only 2.3%, see columns “***J***” in Table 2. Predictions regarding breast cancer subtype deviate by about 4.7%, see column “***J***” of Table 4.

Differences between fRMA and GCRMA seem more pronounced: Comparison of single values shows distinct trends, most probably reflecting probe-dependent bias correction by GCRMA, see Figure 8. Surprisingly, discordance in biomarkers remains low: 1.8% for hormone receptors and 4.3% for breast cancer subtypes, see columns “***K***” in Table 2 and 4.

#### 3.3.4. Only Seemingly a Batch Correction: PLIER

PLIER, being implemented in R, for technical reasons cannot handle all 3753 samples in one bunch. But even when normalizing single studies (token “PLIER R Single”), PLIER seems to suppress batch effects drastically, see panel (c) in Figure 3. Seemingly, its probe set-specific bias correction also reduces batch effects between studies: ***q***_0.80_ exhibits fairly the same variability within studies as seen between studies. This feature of PLIER hints to poor performance regarding biomarkers, if single pipelines are evaluated (see chapter 3.6).

### 3.4. Performance of Individual Pipelines

Up to now, we have compared pairs of pipelines, now we evaluate single pipelines: Following normalization via each pipeline, logistic regression is trained as described in our previous work [50], using a single cut-point, rendering no cases undecided. Predictions from gene expression for receptor status are then checked against the golden standard, IHC. Better concordance is considered to root in superior preprocessing (normalization et al.), allowing to build more adequate biomarkers.  Table 5 and Table S3 show concordance between receptor prediction and golden standard, based on percent misclassification and Matthews correlation coefficient [57], respectively.

### 3.5. Explicit Batch Correction

As the first procedure to eliminate batch effects after normalization, we considered ComBat [12], being implemented in R within the SVA-package. ComBat looks promising, since it draws on empirical Bayes methods [58] and evidence approximation [59]. However, a fundamental precondition for its applicability is not fulfilled by our data: We do not work with two groups of patients (e.g., disease versus control), for which ComBat is designed, but consider tumor patients only (one group). We nevertheless tested ComBat, defining 38 (study-)batches according to different GSE-numbers. As a result, applying ComBat after RmaMSingle worsens receptor status prediction from 10.3% to 18.2% misclassification, see Table 6 and Table S4. About the same effect is seen after global normalization (results not shown). This finding is no surprise, after all preconditions for the application of ComBat are not fulfilled by our data.

As a second procedure to eliminate batch effects after normalization, we considered surrogate variable analysis (SVA), [13, 32, 42, 43]. SVA accepts as input models for “variables of interest” and suspected “batches” (jointly labelled “ViBatch” in the following, see Table 6 and Table S4). SVA first corrects for all effects explainable by these input models and on top of that estimates how many additional surrogate variables (“#SurrVar” in Table 6 and Table S4) could explain residual variability. SVA is first applied to a training set (given as “#samples for training” in Table 6 and Table S4). The result of training is subsequently fed into the correction algorithm, “frozenSVA”, which can be applied to any set of samples (test set), in our case all 3753 samples. We tested SVA with three different input models for variables of interest and batch information (ViBatch) in the following steps, see Table 6:

1. SVA with ViBatch = {ER, GSE} was trained on 3014 samples (with IHC-ER estimates available), with the ER declared as the only variable of interest: SVA estimates 9 surrogate variables when applied to data normalized for single studies (RmaMSingle), see Table 6.

Based on that, we applied fSVA and obtained batch-corrected corrected expression values for all 3753 samples. Our odds-prediction algorithm [17, 50] was applied, based on gene and cogene of each receptor (ER, PGR, HER2). Unexpectedly, prediction quality worsened: percent misclassified receptors increased from 10.7% (average for RmaMSingle) to 21.3%, see Table 6. Note that for each receptor, percent misclassification can be evaluated only for samples with available IHC estimate (numbers given as “#samples for testing” in Table 6).

After global normalization (RmaMGlobal), SVA estimated 8 surrogate variables to be relevant. Based on that, fSVA was applied to all 3753 samples, and the odds-prediction performed. Again misclassification increased, but only slightly—from 9.7% to 10.0%, see Table 6

(2) Next, we trained SVA with ViBatch = {ER, HER2, GSE} on 2291 samples (with IHC-ER and -HER2 available), and the IHC-estimate of ER and HER2 as variables of interest. We did not include PGR as variable of interest since it is physiologically highly correlated with ER, and SVA—by design—should not be fed with models including correlated variables. HER2 is but a variable of interest, uncorrelated with both others. SVA estimates 5 and 9 surrogate variables for RmaMSingle- and RmaMGlobal-normalized data, respectively, see Table 6

Applying fSVA after RmaMSingle again worsens prediction (10.7% to 15.0% misclassification on average). As opposed to this, fSVA after RmaMGlobal slightly improves prediction on average (9.8% to 9.5%). It is noteworthy that PGR performs exceptionally weak in this setting: misclassification rises from 14.1% to 24.2% for fSVA after RmaMSingle. And even after RmaMGlobal, some worsening is noticeable (from 12.5% to 13.4%).

We suspected that the bad performance of PGR-prediction might root in the fact that it was not declared a variable of interest—despite good reasons for that (its correlation with ER). To clarify, we performed an additional test

(3) SVA was trained with ViBatch = {ER, PGR, HER2, GSE}, i.e., the maximum model, on 1825 samples with all 3 IHC estimates available. After RmaMSingle, it estimated 8 surrogate variables, and after fSVA the overall performance of the odds-prediction method worsened even more, from 10.7% to 25.1%. Not even PGR-prediction itself benefitted from including PGR as variable of interest. It rather worsened (24.2% to 27.5% misclassification).

After global normalization, SVA estimated 10 surrogate variables, and fSVA yielded a marginal improvement in classification (decreases misclassification from 9.7% to 9.5% on average)

Batch correction was performed alternatively with ComBat and also with surrogate variable analysis (SVA), in two modes. #SurrVar gives the number of surrogate variables, as estimated by the SVA-package. Values show percent misclassification of hormone receptors as predicted by the odds-model (single cutoff) versus IHC. Note that the single cutoff creates to 2 levels (pos, neg). Each mode of SVA was performed after both, RMA-normalization GSE-wise (RmaMSingle) and globally over all GSEs in one bunch (RmaMGlobal). Note that different numbers of samples for training root in the fact that ER, PGR, and HER2 were available only for respective subsets of samples (# samples). GSE is, of course, always known. For ease of reference, we repeat here the results for RmaMSingle RmaMGlobal (bold entries) already shown in Table 5.

## 4. Discussion

### 4.1. Batch Corrections

Above results indicate for the prediction of receptor status out of 2 genes (per receptor):

1. Performing batch correction (ComBat or SVA) after single RMA normalization increases the percentage of misclassified patients from 10.7 to 15–27%, depending on the particular mode of batch correction, see Table 6
2. Performing SVA after global RMA normalization leaves the percentage of misclassified patients almost unchanged (9.5 to 10.0%), as compared to 9.7% with global RMA normalization, see Table 6

We conclude that “global normalization” is to be preferred against “single normalization plus batch correction”, at least for the prediction models we have evaluated. We have to formulate our conclusion with caution, since it may be possible that batch correction (ComBat and SVA) provides significant benefit for the establishment of new markers (e.g., producing sorted lists of fold changes), as indicated in the literature. It is important to note that our markers are very “small” (just two genes for each receptor), and our conclusion pertains to such cases only. As opposed, batch corrections are designed to correct whole expression profiles, across many genes. Hence “broad markers”—involving many genes—may well benefit.

Further research will be necessary to exactly specify the domain for the beneficial application of batch corrections. For the markers considered here, batch corrections (ComBat, SVA) did not prove very helpful and we hence focus on comparing normalization methods against each other in the following conclusions.

### 4.2. Assembling the “Optimum Pipeline”

R- and MATLAB- implementations of RMA differ very little, as compared to the vast difference of both (compared) to MAS5. We conclude that differences seen in statistical measures do not necessarily corrupt biomarker usability.

R is a free and powerful software framework and hence most widely used for statistical analysis. However, numerous bioinformatics algorithms have also been implemented in MATLAB, a commercial and fairly costly platform. We have compared the results of the same algorithms, implemented in R and MATLAB, respectively.

What truly remains to be selected is between two flavors:

1. RMA, fRMA, or IRON (all these perform blunt, overall bias correction) or
2. GCRMA and PLIER (performing probe-specific bias correction)

RMA in R has limitations for large datasets. These can de facto only be handled in MATLAB—the latter being not really an option for the majority of users due to costs.

IRON and fRMA do not differ a lot. For making a choice, we remind that IRON computes a reference chip out of those chips submitted—and hence results for a given sample always depend on the remaining set of samples submitted. Opposed to this works fRMA, it draws from information out of GEO, irrespective of particular data submitted. Hence, we prefer fRMA against IRON.

Both, GCRMA and PLIER, compute probe-specific affinity estimates—not from all of GEO but rather from the set of samples submitted for analysis. Unfortunately, PLIER suffers from its inability to handle a large number of samples (seemingly due to implementation weaknesses in R, similar to RMA). This precludes global normalization of all studies, and one has to retreat to normalization of single studies. Despite this drawback, results look promising, as long as one considers nothing but ***q***_0.80_: PLIER seemingly eliminates most of the study bias, see Figure 10. However, PLIER will not pass the final criterion in our selection pathway: Even though ***q***_0.80_ fails to reveal any study bias, receptor status diagnostics is worse than with any other pipeline considered. Hence, PLIER cannot be the pipeline to conclusively suggest.

GCRMA also reduces probe-specific bias and should therefore be a promising candidate in a final selection.

### 4.3. Comparison of Top Pipelines

**Figure 16:**
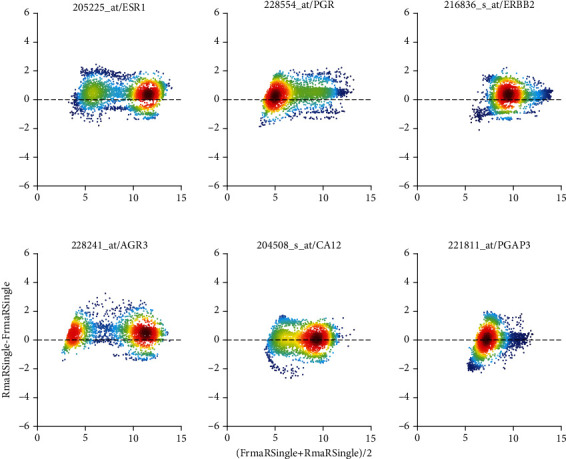
fRMA versus RMA R Single shown as Bland-Altman plot (comparison “*M*”). For details regarding panels and axes, see caption of Figure 7.

In our final lap, we display the “derby” fRMA against “GCRMA R global” (comparison “***K***”) and compare each of these two against the dinosaur “RMA R single”, being the pipeline most frequently used up to now throughout the community, see Figure 6 and Figure 16. For pairwise comparisons on probe-set level, see comparison “***K***”, Figure 8. The differences in effect on biomarkers are shown in Columns “***M***” and “***N***” of Table 3 to Table S1.

GCRMA subtracts an estimate of positive bias in the hybridization of GC-pairs, as already seen in the normalization of single studies; hence, the GCRMA profile is shifted to the left with respect to fRMA (which performs a blunt correction similar to RMA). “Shapes of profiles” are similar, however: fRMA and GCRMA exhibit a shoulder between 6 and 8 of log_2_(expression), see Figure 6, panel (a). Correlation distances have a delta-like peak around 0.02, only very few reach 0.1, see Figure 6, panel (b). Parallel to small correlation distance, disagreement in markers is only 1.8% for receptor status and 4.3% for subtypes (column “***K***” in Tables 3 and 4, respectively). Also, ***q***_0.80_-quantiles reflect a very concordant dependence on samples, see Figure 6 panel (c). The downwards shift of GCRMA is concordant with panel (a).

### 4.4. Top Pipelines versus the Most Common Practice: RMA

The difference between common practice (RmaRSingle) and one of the best choices broadly available today (fRMA) is of special interest: The distance seen in ***q***_0.80_ (Figure 6(c)) corresponds to a horizontal shift in profiles.

Differences regarding biomarkers are ambiguous: Little impact is seen for receptor status prediction (2.8% misclassification on average, see column “***M***” in Table 2). Major impact has to be accepted for breast cancer subtypes (10.5% misclassification on average, see column “***M***” in Table 4). We speculate that biomarkers involving multiple variables (as genefu does) are far more sensitive to details in normalization.

The difference between common practice (RmaRSingle) and “GcrmaRGlobal” (comparison “***N***”) is substantial. Differences in average values indicate a major discrepancy, i.e., a deficiency in at least one of both methods. While profiles are very similar (Figure 6, panel (a)), correlation distances lie around 0.07 (panel (b)), “RMA R Single” leaves us with massive study bias (panel (c)). As opposed, “GCRMA R Global” equalizes study bias to a large extent, at least as far as reflected by ***q***_0.80_. Also regarding biomarkers, differences are substantial: 5.7% average misclassification for receptor status (see column “***N***” in Table 2) and 11.6% for breast cancer subtypes (see column “***N***” in Table 4). Apparent discrepancies are confirmed by the Bland Altman-plot, see Figure 9: Probe-set specific bias correction gives rise to distinct dependencies of difference on size. Concordantly, disagreement in receptor prediction is 3.1% and in subtype classification as much as 11%.

As a final conclusion, we have to concede that common practice (RMA) is far from what deems optimum according to our analysis: “GcrmaRGlobal” or fRMA.

### 4.5. Final Derby

As final showdown, we end up with the “derby”, “GcrmaRGlobal” against fRMA on a probe-set basis, already shown in Figure 8. Now, we draw as final conclusions:

1. Regarding receptor prediction (gene + cogene), the new pipelines (fRMA and GCRMA) have about equal distances towards each other as they both have to the most common practice, RMA single
2. Regarding breast cancer subtypes, the novel methods agree much closer than each of them agrees with RMA single

We may conclude that common practice, i.e., RmaSingle, should be seriously questioned when applied in marker development involving multiple genes.

## 5. Conclusions

The robustness of biomarkers indicates a somewhat different ranking of “distances” between pipelines—as compared to statistical parameters (correlation distance). Both groups of biomarkers seem most sensitive to corrections of bias and background, performed differently by each pipeline.

We conclude that any software intended to compute biomarkers has to be individually calibrated: The particular normalization pipeline used during calibration has to be applied also in the prediction phase. During calibration, we encounter the difficulty that the ground truth, i.e., the true subtype of a patient, is not known for sure (in most cases). Among all pipelines described, several make sense and can be recommended, from others one should rather refrain and some even show peculiar results. In the following, we provide a summary.

### 5.1. Pipelines We Do Not Recommend

To start with, MAS5, although fine regarding concept, yields unfavorable distributions of expression values, precluding reasonable subsequent analysis.

“RMA R Single”, seemingly the “dinosaur” pipeline most frequently used in the bioinformatics community, leaves significant batch effects uncorrected, see Figure S4. Caution is advisable when multiple studies are jointly considered.

ComBat produces dubious outliers and devastates biomarker precision, hence, cannot be recommended.

### 5.2. RMA: When and How to Recommend

RMA performs well, if performed globally over all samples. This is feasible either in its variant fRMA or in MATLAB implementation (pipeline “RMA Matlab global”). Both pipelines render subsequent batch corrections unnecessary.

### 5.3. Batch Corrections: Helpful Only for Multigene Signatures?

Combining multiple studies raises the question of batch correction. Even if statistical measures for single expression values (see Table 3, column “***L***”) seem to advocate for ComBat, biomarkers may be deteriorated (see Table 2, Table S2, and Table 4, column “***L***”). We conclude that markers with few genes (as investigated in this work) do not really benefit from batch correction: Performing batch correction after single normalization worsen results, see Table 6. This is seen for ComBat as well as each mode of SVA investigated. Results are as good as nor after global normalization. On the other hand, global normalization is significantly superior to single normalization. However, performing batch correction after global normalization does not improve results further.

The situation may be totally different for multigene markers, where batch correction may allow for significant improvements. More research seems necessary to develop specific criteria for the usefulness of batch correction in deriving small and broad markers from the same set of studies.

### 5.4. GCRMA: When and How to Recommend

GCRMA yields more usable distribution profiles than RMA does. Beware of differences in results between implementations, however (see Figure S6). Both implementations are capable of global normalization, rendering batch correction unnecessary. Hence, “GCRMA R global” and “GCRMA Matlab global” are two pipelines we recommend.

As a final conclusion, we end up with fRMA and “GCRMA R global” as top pipelines, both rendering subsequent batch correction unnecessary. fRMA has the unique advantage to normalize additional samples in a fairly standardized way: dependence on other samples previously normalized is usually negligible.

### 5.5. Desired Developments

GCRMA performs probe-specific bias correction and seemingly yields more realistic distributions of expression values than RMA does. Even though both implementations can normalize multiple studies, results for single samples still depend on the “community of all other samples” normalized. This specific drawback has been overcome by fRMA. What hence would be desirable is a version of GCRMA with frozen reference. For the majority of users, an implementation in R would be preferable. However, we have to remind that results from R are seriously questioned by those from MATLAB, and quality assurance seems a must for any such new implementation. More research also seems desirable on the conditions for applicability and benefit of batch corrections, ComBat, and SVA.

## Figures and Tables

**Table 1:** Studies in GEO used for present work.

Idx	GSE-ID	City	No. of samples used	Samples in GEO	Criteria for excluding samples					Sample-ID	PMID	Public on
					Controls	Duplicates	Replicates	Others	Comment to excluded others			
1	GSE31448	Marseille	347	357	4			6	Did not pass quality control	1611-1963	22110708	Dec 02, 2011
2	GSE20685	Taipei	327	327						757-1083	21501481	Apr 24, 2011
3	GSE26639	Paris Cedex 05	226	226						1331-1556	22110708	Jun 08, 2011
4	GSE12276	Rotterdam	203	204		1				117-319	19421193	Jun 13, 2009
5	GSE76124	Houston	198	198						3301-3498	25208879 26921331	Dec 18, 2015
6	GSE65194	Paris	153	178	11			14	Cell line	2934-3086	25848952 23700430 23144294	Jan 23, 2015
7	GSE71258	Missouri	128	128						3173-3300	26269605	Dec 16, 2015
8	GSE16446	Toronto	120	120						426-545	21422418 20098429 20189874	Jan 26, 2010
9	GSE88770	Brussels	116	117		1				1215-1330	23028037	Oct 15, 2016
10	GSE23177	Leuven	116	116						3597-3712	21116709	Feb 04, 2011
11	GSE19615	Manhattan	115	115						642-756	20098429	Jan 05, 2010
12	GSE43365	Boston	111	111						2260-2370	na	Jun 03, 2013
13	GSE36771	Auckland	107	107						1992-2098	22564725	Mar 31, 2012
14	GSE58812	Saint Herblain	107	107						2675-2781	25887482	May 19, 2015
15	GSE42568	Dublin	102	121	17			2	Did not pass quality control	2099-2202	23740839	May 26, 2013
16	GSE58984	Brussels	94	94						2782-2875	na	Dec 31, 2016
17	GSE20711	Toronto	88	90	2					1084-1171	21910250	Oct 19, 2011
18	GSE6532	Toronto	87	741				654	Other than breast cancer tissue	30-116	17401012 18498629 20479250	Mar 01, 2007
19	GSE66305	Modena	86	88				2	Did not pass quality control	3087-3172	26245675	Oct 06, 2015
20	GSE48390	New Taipei City	81	81						2466-2546	24098497	Jan 07, 2014
21	GSE18864	Lyngby	75	84			9			567-641	20100965 20098429 20189874	Nov 04, 2009
22	GSE76274	Houston	67	67						3499-3565	25208879 26921331	Dec 23, 2015
23	GSE61304	Singapore	58	62	4					2876-2933	26517092 26474389	May 10, 2015
24	GSE43358	Brussels	57	57						2203-2259	25412710	Nov 21, 2014
25	GSE95700	Taipei City	57	57						3713-3769	28299476	Jun 13, 2017
26	GSE16391	Toronto	55	55						371-425	19573224	Jul 09, 2009
27	GSE29431	Barcelona	46	66	12			8	Did not pass quality control	1557-1610	na	Dec 22, 2011
28	GSE12777	South San Francisco	51	51						320-370	19567590 21673316	Jan 12, 2009
29	GSE58792	New York	51	51						2624-2674	25190728	Jul 01, 2014
30	GSE46222	Washington	48	49				1	CEL not processable in R	2371-2418	na	Apr 15, 2015
31	GSE47389	Rotterdam	47	47						2419-2465	23993096	Nov 01, 2013
32	GSE22035	Saint-Cloud	43	43						1172-1214	21209903	Jan 10, 2011
33	GSE48905	Hørsholm	42	42						2547-2588	24505287	Jan 08, 2014
34	GSE50567	Gliwice	35	41	6					2589-2623	21196292	Sep 05, 2013
35	GSE87007	Brussels	31	31						3566-3596	28566333	Jan 01, 2017
36	GSE5460	Boston	29	129		98		2	CELs not processable in R	1-29	18297396	May 26, 2007
37	GSE27120	Brussels	28	79	3			48	No receptor status	1964-1991	22235100	Mar 15, 2012
38	GSE18728	Seattle	21	61			40			546-566	20012355	Oct 30, 2009
Total:			3753	4698	59	100	49	737				

**Table 2:** Impact of data processing pipeline on percent misclassification of hormone receptor status.

		Hormone receptor status prediction: percent misclassification													
Receptor	#	**A**	**B**	**C**	***D***	***E***	***F***	***G***	***H***	***I***	***J***	***K***	***L***	***M***	***N***
*ER*	3	**5.4**	**3.3**	**3.7**	1.6	3.8	0.3	1.4	2.9	0.8	1.5	1.6	15.6	2.8	2.7
*PGR*	3	**10.9**	**7.9**	**8.1**	4.8	8.8	0.7	4.8	6.8	2.2	2.7	2.0	21.6	7.1	7.3
*HER2*	3	**8.0**	**6.5**	**5.3**	4.7	7.1	0.4	3.1	6.7	2.2	2.5	2.0	11.7	6.4	7.0
Average		**8.1**	**5.9**	**5.7**	3.7	6.6	0.4	3.1	5.4	1.8	2.3	1.8	16.3	5.4	5.7

**Table 3:** Correlation distance between pipelines.

	Correlation distance between pipelines													
	**A**	**B**	**C**	***D***	***E***	***F***	***G***	***H***	***I***	***J***	***K***	***L***	***M***	***N***
	**Mas5RSingle PlierRSingle**	**Mas5RSingle RmaRSingle**	**PlierRSingle RmaRSingle**	RmaRSingle GcrmaRSingle	PlierRSingle GcrmaRSingle	RmaRSingle RmaMSingle	GcrmaMSingle GcrmaRSingle	RmaMGlobal RmaMSingle	GcrmaMGlobal GcrmaRGlobal	FrmaRSingle IronGlobal	FrmaRSingle GcrmaRGlobal	GcrmaRGlobal GcrmaRSingleCombat	FrmaRSingle RrmaRSingle	RmaRSingle GcrmaRGlobal
Mean	**0.153**	**0.110**	**0.190**	0.072	0.205	0.001	0.064	0.012	0.065	0.022	0.067	0.021	0.021	0.075
Median	**0.147**	**0.103**	**0.181**	0.067	0.198	0.001	0.057	0.008	0.060	0.021	0.063	0.017	0.018	0.070
***q***_0.15_	**0.129**	**0.090**	**0.155**	0.054	0.173	0.001	0.043	0.005	0.054	0.018	0.054	0.011	0.016	0.058
***q***_0.85_	**0.175**	**0.132**	**0.221**	0.093	0.235	0.002	0.089	0.020	0.076	0.024	0.079	0.028	0.025	0.096

**Table 4:** Impact of data processing pipelines on 6 algorithms for breast cancer subtype classification: Percent misclassification for pairwise comparisons between pipelines.

		Breast cancer subgroup prediction: percent misclassification													
Algorithm	#	**A**	**B**	**C**	***D***	***E***	***F***	***G***	***H***	***I***	***J***	***K***	***L***	***M***	***N***
scmgene	4	**49.9**	**31.1**	**58.5**	9.7	59.6	1.1	8.1	10.8	6.2	4.0	5.8	23.6	11.1	12.0
scmod1	4	**21.5**	**18.2**	**16.1**	13.2	20.7	2.0	6.1	18.4	2.5	4.3	2.4	23.4	18.1	17.9
scmod2	4	**22.5**	**20.0**	**16.8**	11.3	18.7	1.7	5.1	18.1	1.6	5.3	2.5	23.2	18.3	18.9
pam50	5	**16.8**	**7.6**	**20.9**	4.8	20.9	1.0	5.6	5.6	4.8	4.7	4.7	20.4	4.8	6.0
ssp2006	5	**10.9**	**10.6**	**11.1**	8.4	13.9	1.5	7.6	5.9	4.5	5.8	4.9	22.7	6.4	8.6
ssp2003	5	**12.1**	**9.5**	**15.5**	7.3	18.8	1.8	7.9	4.6	4.6	4.4	5.4	24.0	3.9	6.0
Average		**22.3**	**16.2**	**23.2**	9.1	25.4	1.5	6.7	10.6	4.0	4.7	4.3	22.9	10.5	11.6

**Table 5:** Performance of receptor status prediction after different normalization pipelines.

			Hormone receptor status prediction: percent misclassification against IHC												
Samples		#	RmaMGlobal	GcrmaMGlobal	FrmaRSingle	IronGlobal	Mas5RSingle	PlierRSingle	RmaRSingle	RmaMSingle	GcrmaMSingle	GcrmaRGlobal	GcrmaRSingle	GcrmaRSingleCombat	Without normalization
*ESR1*	3014	2	8.0	8.0	8.3	8.9	9.0	11.5	10.3	8.5	10.1	7.9	9.3	19.9	12.0
*PGR*	2170	2	12.5	12.6	12.4	14.2	14.5	17.6	15.4	14.1	15.6	12.6	14.9	18.8	20.2
*HER2*	2443	2	8.5	8.5	8.8	9.8	9.0	11.1	9.7	9.6	9.7	8.8	9.9	15.4	10.9
Average			9.7	9.7	9.8	11.0	10.8	13.4	11.8	10.7	11.8	9.8	11.3	18.0	14.4

# denotes the number of levels for predictor (positive, negative); numbers are percentages of patients with predictions from gene expression different from the golden standard, IHC. The last column gives values for the use of raw, nonnormalized data from CEL-files. Results in terms of Matthews correlation coefficient are given in Table S3.

**Table 6:** Batch correction after RMA normalization: percent misclassification in the prediction of hormone receptors.

	# samples for testing	no batch correction		ComBat: ViBatch: {GSE}	fSVA: ViBatch: {ER, GSE} 3014 samples for training		fSVA: ViBatch: {ER, HER2, GSE} 2291 samples for training		fSVA: ViBatch: {ER, PGR, HER2, GSE} 1825 samples for training	
		**RmaMSingle**	**RmaMGlobal**	RmaMSingle	RmaMSingle	RmaMGlobal	RmaMSingle	RmaMGlobal	RmaMSingle	RmaMGlobal
*# SurrVar*		**—**	**—**	—	9	8	5	9	8	10
*ESR1*	3014	**8.5**	**8.0**	19.6	20.7	8.1	10.5	8.0	23.6	8.1
*PGR*	2170	**14.1**	**12.5**	19.0	31.5	13.2	24.2	13.4	27.5	13.3
*HER2*	2443	**9.6**	**8.5**	15.1	11.6	8.7	10.3	7.0	24.2	7.1
**Average**		**10.7**	**9.7**	**17.9**	**21.3**	**10.0**	**15.0**	**9.5**	**25.1**	**9.5**

## Data Availability

All data were downloaded from Gene Expression Omnibus. Expression data normalized with several methods discussed in this work are provided for download in supplementary materials.

## Supplementary Materials

Supplementary Materials Figure S1: MAS5 versus PLIER, applied to single studies (Comparison “A”) via Bland-Altman plots of log2-expression values. Figure S2: MAS5 versus RMA, applied to single studies (Comparison “B”) via Bland-Altman plots of log2-expression values. Figure S3: GcrmaRSingle vs. PlierRSingle (Comparison “E”) via Bland-Altman plots of log2-expression values. Figure S4: differences in RMA and GCRMA normalization between R and MATLAB (Comparisons “F” and “G”). Figure S5: RMA implemented in R versus MATLAB (Comparison “F”) via Bland-Altman plots of log2-expression values. Figure S6: GCRMA implemented in R versus MATLAB (Comparison “G”) via Bland-Altman plots of log2-expression values. Table S1: impact of data processing pipelines on 6 algorithms for breast cancer subtype classification: Cohen's kappa for pairwise comparisons between pipelines. Table S2: impact of data processing pipeline on hormone receptor estimates. Table S3: performance of receptor status prediction after different normalization pipelines. Table S4: batch correction after RMA normalization: Matthews correlation coefficient for the prediction of hormone receptors. Table S5: runtimes and characteristics of selected normalization procedures.
