# Supplementary material for: Microarray Normalization Revisited for Reproducible Breast Cancer Biomarkers
Source: Biomed Res Int. 2020 Aug 6;2020:1363827. doi: 10.1155/2020/1363827 (PMC7428878; doi:10.1155/2020/1363827)
Supplement: Supplementary Materials — Figure S1: MAS5 versus PLIER, applied to single studies (Comparison “A”) via Bland-Altman plots of log2-expression values. Figure S2: MAS5 versus RMA, applied to single studies (Comparison “B”) via Bland-Altman plots of log2-expression values. Figure S3: GcrmaRSingle vs. PlierRSingle (Comparison “E”) via Bland-Altman plots of log2-expression values. Figure S4: differences in RMA and GCRMA normalization between R and MATLAB (Comparisons “F” and “G”). Figure S5: RMA implemented in R versus MATLAB (Comparison “F”) via Bland-Altman plots of log2-expression values. Figure S6: GCRMA implemented in R versus MATLAB (Comparison “G”) via Bland-Altman plots of log2-expression values. Table S1: impact of data processing pipelines on 6 algorithms for breast cancer subtype classification: Cohen's kappa for pairwise comparisons between pipelines. Table S2: impact of data processing pipeline on hormone receptor estimates. Table S3: performance of receptor status prediction after different normalization pipelines. Table S4: batch correction after RMA normalization: Matthews correlation coefficient for the prediction of hormone receptors. Table S5: runtimes and characteristics of selected normalization procedures. [file 1363827.f1.pdf]

7 Supplementary Material

Supplementary text and material is contained in https://meduniwien.ac.at/MicroarrayNormalization/MicroarrayNormalizationRevisited.zip

It contains:

- Supplementary text, tables and figures (SupplText.pdf)
- Clinical data, e.g. IHC receptor status, come with expression values as part of the 'characteristics' according to GEO terminology. Precise and secure selection of series and samples, in particular those who provide most comprehensive receptor status, is essential for high quality meta-studies. We provide a tool tailored for this purpose, geoSeriesSummarization, see the sub folder "GeoSeriesSummarization".
  It holds a description for usage (_readme_GeoSeriesSelectionAndSummarization.txt), documentation (index.html in subfolder 'doc'), the java-code (geoSeriesSummarization.jar), an example-input file (ExampleGseSet.xlsx) and a corresponding result file (exampleOut.xlsx).
- The workflow for finding duplicate samples, the normalized expression data, sample description with receptor status (_readme_checkDuplicatesAndNormalization.txt), a snippet of R-code (source.import.R).

7.1 Distance between samples that have been normalized through different pipelines

For high dimensional data (e.g. 54675 probe-sets associated with 23035 genes for the U133 Plus 2.0 chip), most conventional measures of distance fail: Applying Euclidean (L2) norm, a minority of probes yields the major contribution to overall distance between two samples. Hence, L2 cannot be considered representative. The L1-norm (city block) is slightly less sensitive. However, between different normalizations significant additive offsets are frequently seen and hence only samples normalized within the same bunch should be compared via L1.

In our case measures of correlation are more adequate, in particular the 'correlation distance' between pipelines, π_1 and π_2: $d_{\pi_1,\pi_2}(i) = 1 - \rho_{\pi_1,\pi_2}(i)$. A given sample, *i*, is normalized by 2 different pipelines, and $\rho_{\pi_1,\pi_2}(i)$ is the Pearson correlation coefficient, computed over all probe-sets within sample *i*. We average correlation distance $d_{\pi_1,\pi_2}(i)$ over all samples and – in addition – provide quantiles to estimate the 'distance' between normalization pipelines, π_1 and π_2, see **Table 3**.

7.2 Probe-wise comparison between MAS5, RMA and PLIER

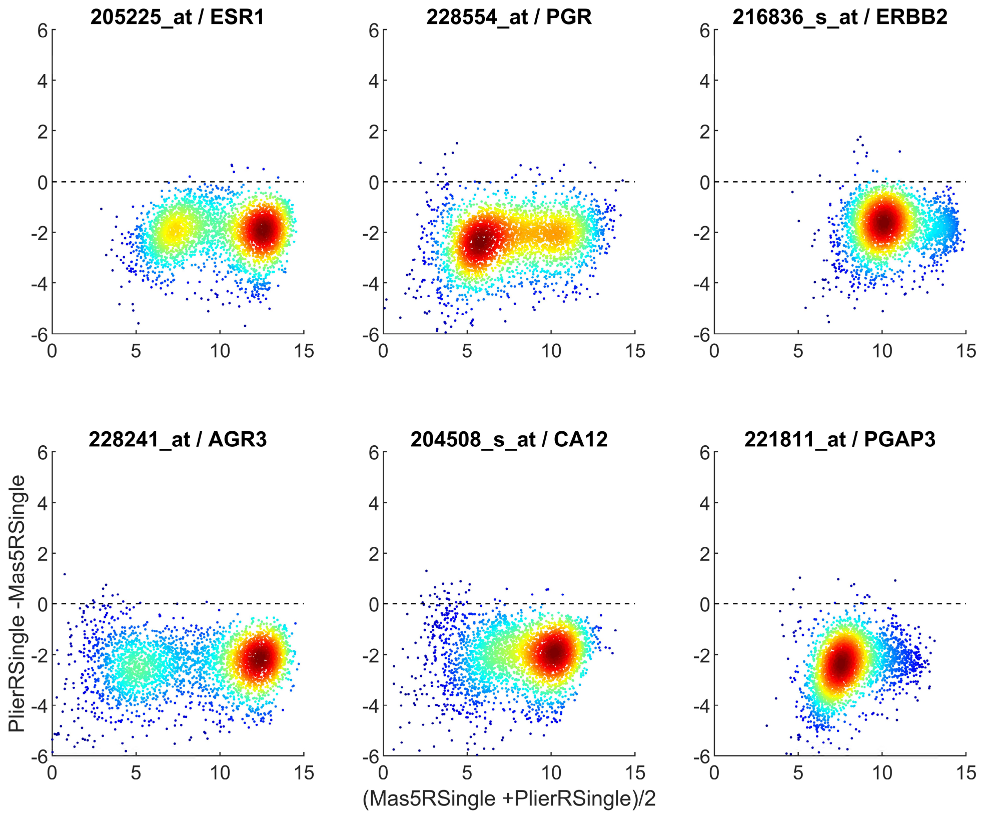

Figure S1: MAS5 versus PLIER, applied to single studies (Comparison '*A*') via Bland-Altman plots of $\log_2$-expression values. For details regarding panels and axes see caption of **Fig. 7**

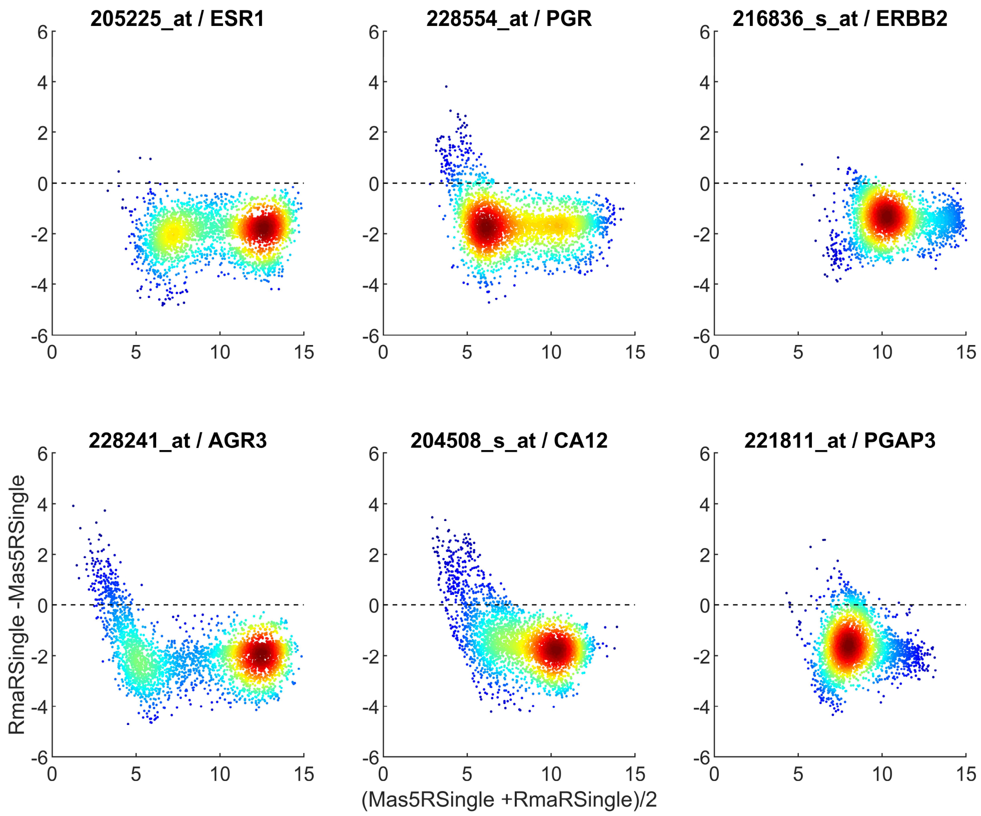

Figure S2: MAS5 versus RMA, applied to single studies (Comparison '*B*') via Bland-Altman plots of log_2-expression values.
For details regarding panels and axes see caption of **Fig. 7**. MAS5 results are systematically lower than RMA (general bias). Distinct trends are visible, in particular for low expression values. They most probably result from probe-specific bias correction performed by MAS5 in contrast to RMA, performing not more than an average background correction.

7.3 Probe-wise comparison between GCRMA and PLIER

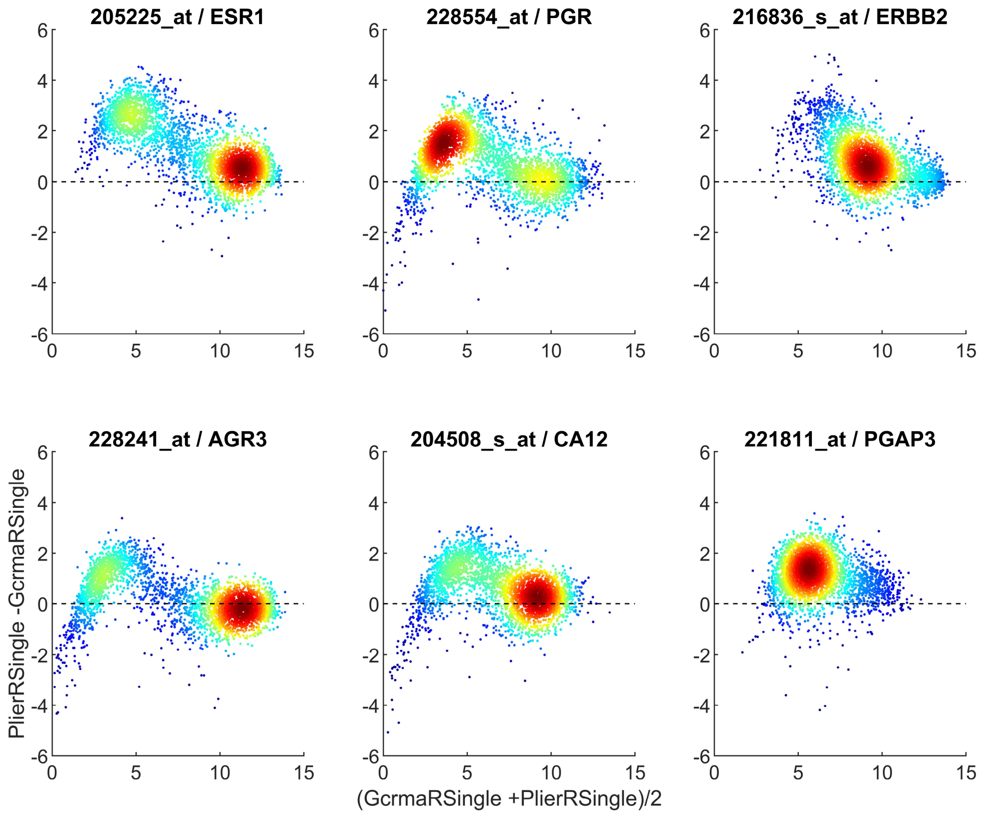

Figure S3: GcrmaRSingle vs. PlierRSingle (Comparison 'E') via Bland-Altman plots of log^{2}-expression values.
For details regarding panels and axes see caption of **Fig. 7**.

7.4 R versus Matlab

We compare

- **RMA** implemented in R versus MATLAB (tokens 'RmaRSingle' vs. 'RmaMSingle', Comparison '***F***') and
- **GCRMA** in R versus MATLAB (tokens 'GcrmaRSingle' vs. 'GcrmaMSingle', Comparison '***G***').

For an overview see **Figure S4**, for details **Figure S5** and **Figure S6**.

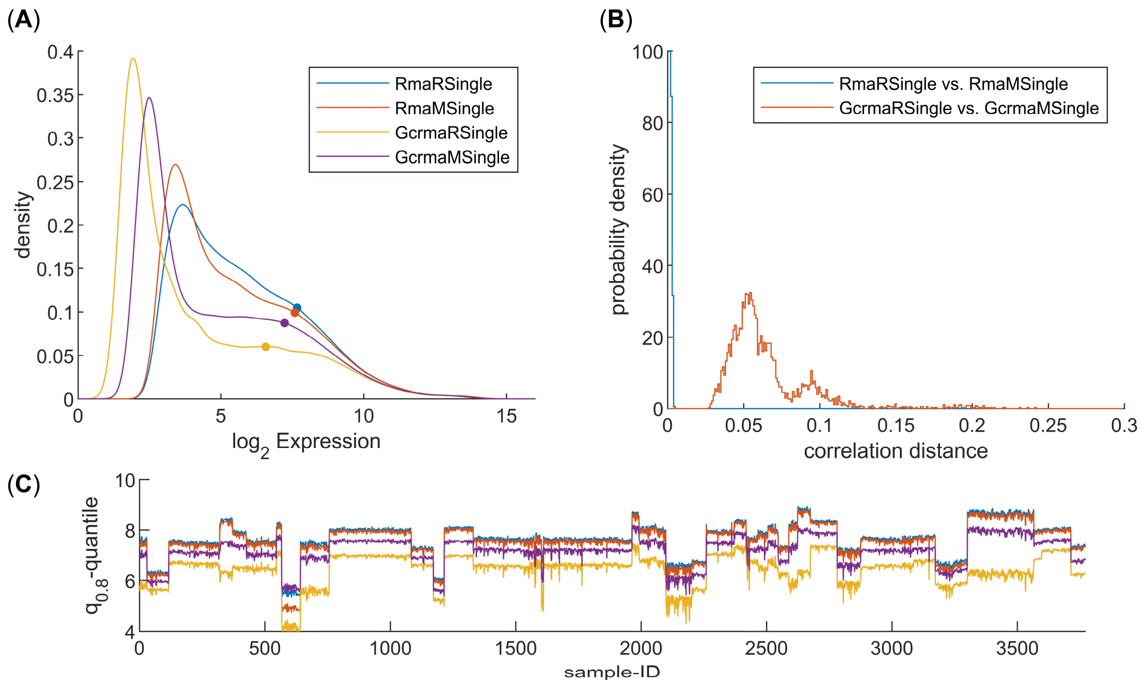

Figure S4: Differences in RMA and GCRMA normalization between R and MATLAB (Comparisons '*F*' and '*G*').

Panel (**A**): distribution profiles of log_2-RMA normalized expression values.

Panel (**B**): Histogram of correlation distances of log_2-normalized expression values between pairs of pipelines.

Panel (**C**): For all 54675 probe-sets of each sample the 80% quantile ($\boldsymbol{q}_{0.80}$) is computed and plotted over sample-ID (x-axis). Legend of panel A also applies to panel C

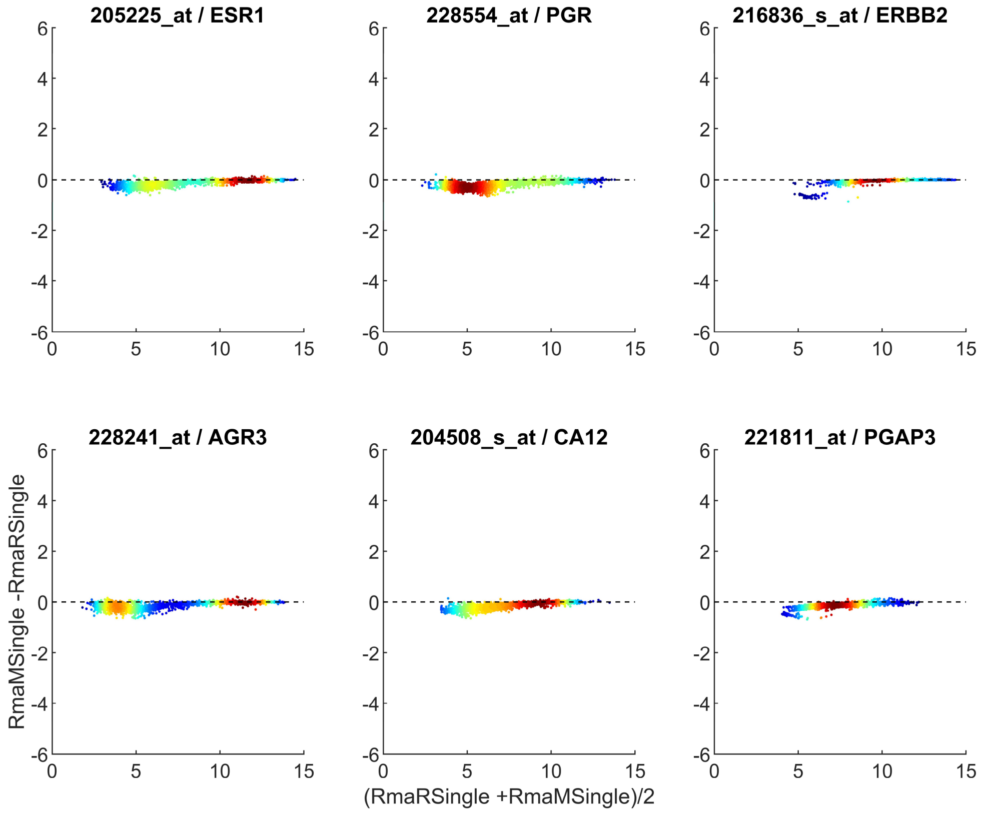

Figure S5: RMA implemented in R versus MATLAB (Comparison 'F') via Bland-Altman plots of log2-expression values.
For details regarding panels and axes see caption of **Figure 7**.Differences in log_2-values around 0.5, as frequently seen in these plots, correspond to ratios RMA_R / RMA_Matlab ≈ $2^{0.5}$ ≈ 1.42.

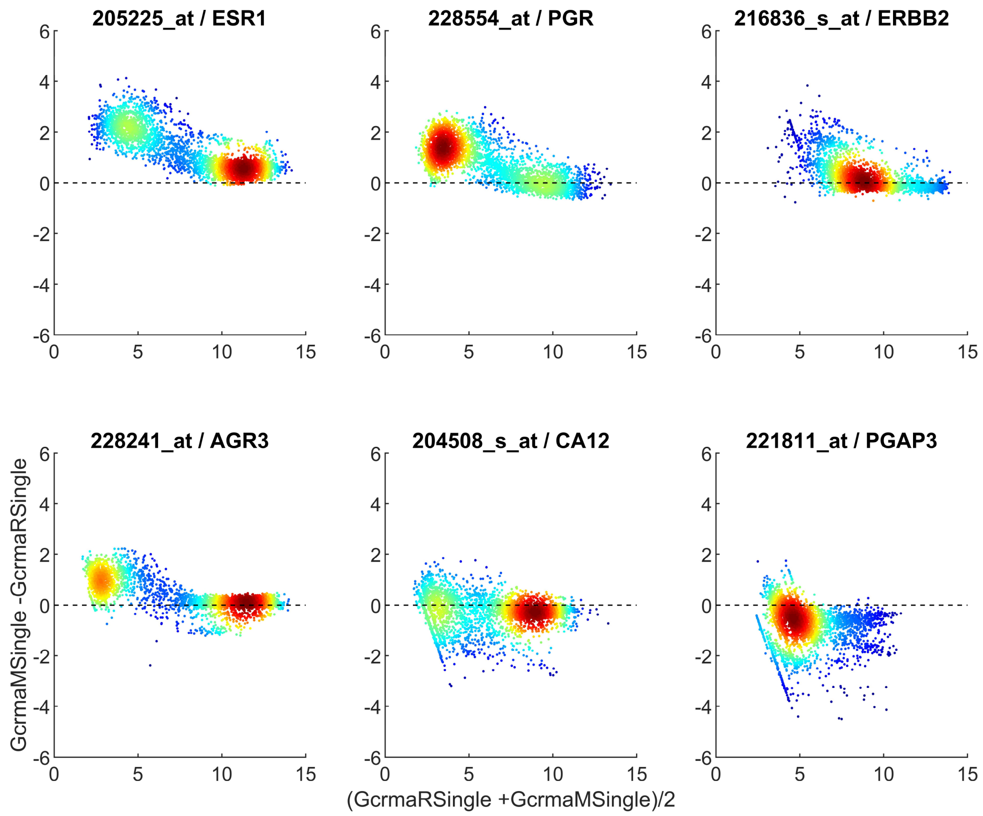

Figure S6: GCRMA implemented in R versus MATLAB (Comparison '*G*') via Bland-Altman plots of log$_2$-expression values.
For details regarding panels and axes see caption of **Figure 7**.

7.4.1 Statistical criteria

The 4 profiles are shown in **Figure S4** panel (**A**). How can such large differences (between implementations of the same algorithm!) be explained? The answer lies most likely in assumptions regarding normalization, different between R and MATLAB. Note however, that these assumptions cannot be forced equal via any parameters accessible to the user. For RMA, differences appear as shifts of normalized values, see the quantiles $\boldsymbol{q}_{0.80}$ in **Figure S4**, panel (**C**). Correlation distances seem small: In a frequency chart they appear as delta-like peaks near zero, see panel (**B**). This close agreement regarding RMA is also reflected by almost zero correlation distance (see column '***F***' in **Table 3**). On the contrary, for GCRMA we observe significant distance, see column '***G***'. This is also reflected in the Bland Altman plots in **Figure S6**.

For RMA, Bland-Altman plots in **Figure S5** indicate that expression values normalized by R exceed those obtained from MATLAB by factors around 1.5, with no systematic trend obvious to the naked eye. Nevertheless, finding factors up to 1.4 between results obtained from different implementations (of the same algorithm) may be alarming, by the way. While profiles change drastically (**Figure S4**, panel (**A**)), correlation distances remain close to zero.

For GCRMA, differences between implementation in R and MATLAB seem even more substantial, see **Figure S6**. In particular for low expression values – but even for medium ones – systematic dependencies are obvious. For the *CA12* gene and for *PGAP3* distinct linear correlations attract attention, possibly resulting from intensity dependent bias correction attempted in GCRMA.

In addition, some warning seems appropriate: The R-implementation of GCRMA is delicate to handle since default parameters have been modified over consecutive software releases, prone to entail difficulties: For large datasets, in need for more computational resources, the parameter 'fast' must paradoxically be set to 'FALSE'. With fast = TRUE in some cases meaningless profiles result, most likely due to background correction being overdone.

7.4.2 Robustness of Biomarkers

When swapping between R and MATLAB implementations of RMA, biomarkers in our test-set remain fairly stable. Only 0.3% – 1.5% of patients switch assignment, see the columns '***F***' in **Table 2** and **Table 4**. In conclusion, for we find:

- Profiles and absolute values differ.
- Sample-distances are close to zero.
- Biomarkers remain almost unaffected.
- Bland Altman-plots (**Figure S5**) do not exhibit remarkable trends.

GCRMA in R versus MATLAB (column '***G***' in **Table 2** and **Table 4**) results in larger discrepancies. In particular, probe-dependent bias correction seems to introduce distinctive, suspicious trends revealed by Bland Altman-plots, see **Figure S6**. Within these larger trends, even more intricate structures – such as linear filaments - are clearly visible, prone to anticipate substantial differences in outcome.

In fact, receptor prediction disagrees in 3.1% of patients, see column '***G***' of **Table 2**. Breast cancer subtype prediction disagrees in 6.7% of patients, see column '***G***' of **Table S1**.

7.5 Stability of biomarkers in terms of kappa

		**Breast cancer subgroup prediction: Cohen's kappa**													
algorithms	#	***A***	***B***	***C***	***D***	***E***	***F***	***G***	***H***	***I***	***J***	***K***	***L***	***M***	***N***
scmgene	4	**0.00**	**0.55**	**-0.41**	0.89	-0.48	0.99	0.91	0.88	0.93	0.96	0.94	0.69	0.87	0.86
scmod1	4	**0.73**	**0.78**	**0.81**	0.85	0.74	0.98	0.93	0.77	0.97	0.95	0.97	0.69	0.78	0.78
scmod2	4	**0.71**	**0.75**	**0.80**	0.87	0.77	0.98	0.95	0.78	0.98	0.94	0.97	0.70	0.78	0.77
pam50	5	**0.80**	**0.92**	**0.74**	0.95	0.74	0.99	0.94	0.94	0.95	0.95	0.95	0.74	0.95	0.94
ssp2006	5	**0.88**	**0.88**	**0.87**	0.91	0.84	0.98	0.92	0.94	0.95	0.94	0.95	0.71	0.93	0.91
ssp2003	5	**0.86**	**0.89**	**0.82**	0.92	0.77	0.98	0.91	0.95	0.95	0.95	0.94	0.69	0.96	0.94

Table S1: Impact of data processing pipelines on 6 algorithms for breast cancer subtype classification: Cohen's kappa for pairwise comparisons between pipelines.
We evaluate six biomarkers computed by algorithms within the R-package 'genefu': Scmgene, scmod1 and scmod2 assign subtypes out of 4 classes, whereas the other 3 assign subtypes out of 5 classes, see column '#'. For each specific comparison (labels '***A***' – '***N***', see **Fig. 1**) between 2 pipelines, we show Cohen's kappa (Fleiss et al., 2003) to quantify inter-rater agreement.

		**Hormone receptor status prediction: Cohen's kappa**													
receptor	#	***A***	***B***	***C***	***D***	***E***	***F***	***G***	***H***	***I***	***J***	***K***	***L***	***M***	***N***
ER	3	**0.90**	**0.94**	**0.93**	0.97	0.93	1.00	0.98	0.95	0.98	0.97	0.97	0.74	0.95	0.95
PGR	3	**0.82**	**0.87**	**0.87**	0.92	0.86	0.99	0.92	0.89	0.96	0.96	0.97	0.66	0.89	0.88
HER2	3	**0.80**	**0.84**	**0.87**	0.88	0.83	0.99	0.92	0.83	0.94	0.94	0.95	0.71	0.84	0.83

Table S2: Impact of data processing pipeline on hormone receptor estimates.
Pairwise comparisons ('***A***' - '***N***') between pipelines, see **Fig. 1**. Note that each estimate may assume one of 3 states (positive, negative, indefinite) as indicated in column #. Numbers give Cohen's kappa (κ) (Fleiss et al., 2003) to quantify inter-rater agreement. Note that this table comprehensively shows all comparisons between pipelines discussed in this work (see **Fig. 1**).

7.6 Performance of receptor status prediction in terms of Matthews correlation coefficient

			**hormone receptor status prediction via gene expression: Matthews correlation**												
	samples	#	RmaMGlobal	GcrmaMGlobal	FrmaRSingle	IronGlobal	Mas5RSingle	PlierRSingle	RmaRSingle	RmaMSingle	GcrmaMSingle	GcrmaRGlobal	GcrmaRSingle	GcrmaRSin-gleCombat	without normalization
ESR1	3014	2	0.92	0.84	0.83	0.90	0.90	0.87	0.89	0.91	0.89	0.84	0.90	0.75	0.86
PGR	2170	2	0.87	0.74	0.75	0.83	0.83	0.79	0.82	0.86	0.82	0.74	0.83	0.77	0.75
HER2	2443	2	0.91	0.77	0.77	0.89	0.90	0.88	0.89	0.90	0.89	0.76	0.89	0.82	0.88

Table S3: Performance of receptor status prediction after different normalization pipelines. # denotes number of levels for predictor (positive, negative), values give Matthews correlation for agreement between predictions from gene expression versus golden standard, IHC. Last column gives values for use of raw, non-normalized data from CEL-files.

7.7 Batch-correction after RMA normalization: Matthews correlation coefficient for the prediction of hormone receptors

		no batch correction		ComBat: ViBatch: {GSE}	fSVA: ViBatch: {ER, GSE} 3014 samples for training		fSVA: ViBatch: {ER, HER2, GSE} 2291 samples for training		fSVA: ViBatch: {ER, PGR, HER2, GSE} 1825 samples for training	
	# samples for testing	**RmaMSingle**	**RmaMGlobal**	RmaMSingle	RmaMSingle	RmaMGlobal	RmaMSingle	RmaMGlobal	RmaMSingle	RmaMGlobal
# SurrVar		-	-	-	9	8	5	9	8	10
ESR1	3014	**8.83**	**0.84**	0.60	0.57	0.84	0.78	0.83	0.52	0.83
PGR	2170	**0.71**	**0.75**	0.61	0.35	0.73	0.50	0.73	0.43	0.73
HER2	2443	**0.74**	**0.77**	0.58	0.68	0.80	0.72	0.81	0.30	0.81

Table S4: Batch-correction after RMA normalization: Matthews correlation coefficient for the prediction of hormone receptors. Values give Matthews correlation coefficient for agreement between predictions from gene expression versus golden standard, IHC.

7.8 Runtimes of selected normalization methods

Runtimes of selected normalization methods have been evaluated and are given in **Table S5**. Note that execution times also depend on available memory and **Table S5** provides only estimates for the specific configuration used in this work.

	RmaMGlobal	GcrmaMGlobal	FrmaRSingle	GcrmaRGlobal
**Environment**	Matlab	Matlab	R	R
**handles GC-hybridisation**	no	yes	no	yes
**runtime [h] (incl. loading CELs)**	5:07	3:14	12:06	45:32
**time for loading CELs [h]**	1:58	2:18	0:12	0:12
**can process zipped CELs**	no	no	yes	yes
**able to process all (3753) samples at once**	yes	yes	no	yes
**floating point precision**	single	single	double	double
**results depend on seed**	no	yes	no	no
**Availability**	license	license	free	free
**support**	professional	professional	community	community
**batch effect reminescants**	very small	small	very small	small
**designed to handle single samples**	no	no	yes	no
**interface to Bioconductor**	no	no	yes	yes
**tuning parameters required**	no	no	no	yes

Table S5: Runtimes and characteristics of selected normalization procedures. All test were run on a server with 2 Intel® Xeon® processors E5-2690 (each having 8 cores with a frequency of 2.9GHz) and 256GB RAM under Ubuntu 18.04. Normalization FrmaRSingle was performed in 2 batches and runtimes added. Each of the other three normalizations was able to handle all 3753 samples. Times given include loading and preprocessing the data (CELs). In addition, loading times are given. Note that GcrmaMGlobal uses Monte Carlo methods and hence results depend on initialization seed.
